# Convergence of monosynaptic inputs from neurons in the brainstem and forebrain on parabrachial neurons that project to the paraventricular nucleus of the thalamus

**DOI:** 10.1007/s00429-022-02534-6

**Published:** 2022-07-15

**Authors:** Gilbert J. Kirouac, Sa Li, Shuanghong Li

**Affiliations:** 1 Department of Oral Biology, Dr. Gerald Niznick College of Dentistry, Rady Faculty of Health Sciences, University of Manitoba, 780 Bannatyne Avenue, Winnipeg, MB R3E 0W2 Canada; 2 Departments of Psychiatry and Human Anatomy and Cell Sciences, Max Rady College of Medicine, Rady Faculty of Health Sciences, University of Manitoba, Winnipeg, MB R3E 0W2 Canada

**Keywords:** Thalamus, Rabies, Viral tracing, Homeostasis, Catecholamine, Arousal

## Abstract

The paraventricular nucleus of the thalamus (PVT) projects to areas of the forebrain involved in regulating behavior. Homeostatic challenges and salient cues activate the PVT and evidence shows that the PVT regulates appetitive and aversive responses. The brainstem is a source of afferents to the PVT and the present study was done to determine if the lateral parabrachial nucleus (LPB) is a relay for inputs to the PVT. Retrograde tracing experiments with cholera toxin B (CTB) demonstrate that the LPB contains more PVT projecting neurons than other regions of the brainstem including the catecholamine cell groups. The hypothesis that the LPB is a relay for signals to the PVT was assessed using an intersectional monosynaptic rabies tracing approach. Sources of inputs to LPB included the reticular formation; periaqueductal gray (PAG); nucleus cuneiformis; and superior and inferior colliculi. Distinctive clusters of input cells to LPB-PVT projecting neurons were also found in the dorsolateral bed nucleus of the stria terminalis (BSTDL) and the lateral central nucleus of the amygdala (CeL). Anterograde viral tracing demonstrates that LPB-PVT neurons densely innervate all regions of the PVT in addition to providing collateral innervation to the preoptic area, lateral hypothalamus, zona incerta and PAG but not the BSTDL and CeL. The paper discusses the anatomical evidence that suggests that the PVT is part of a network of interconnected neurons involved in arousal, homeostasis, and the regulation of behavioral states with forebrain regions potentially providing descending modulation or gating of signals relayed from the LPB to the PVT.

## Introduction

The paraventricular nucleus of the thalamus (PVT) is anatomically positioned within neural circuits that regulate behavior (Kirouac 2015) and accumulating evidence shows that this midline thalamic nucleus modulates a variety of appetitive and aversive responses (see recent reviews by Barson et al. 2020; McGinty and Otis 2020; Penzo and Gao 2021; Millan et al. 2017). The connections and mechanisms involved are generating considerable interest in this rather poorly understood component of the behavioral circuits. The PVT is composed of neurons that provide dense projections via highly collateralized axons to the shell of the nucleus accumbens (NAcSh), dorsolateral bed nucleus of the stria terminalis (BSTDL), lateral subregion of the central nucleus of the amygdala (CeL) (Moga et al. 1995; Li and Kirouac 2008; Parsons et al. 2007; Dong et al. 2017; Vertes and Hoover 2008; Li et al. 2021) through which the PVT has been shown to regulate different behaviors (Choi et al. 2019; Penzo et al. 2015; Padilla-Coreano et al. 2012; Do-Monte et al. 2017; Lafferty et al. 2020; Engelke et al. 2021; Dong et al. 2020; Pliota et al. 2018). Afferents from the prefrontal cortex, hypothalamus and the brainstem (Li and Kirouac 2012) are processed by PVT neurons that form a highly divergent efferent projection system to the forebrain (Li et al. 2021).

The PVT has been consistently identified as an area of the brain that becomes active during periods of behavioral arousal (Hsu et al. 2014; Kirouac 2015) and a number of studies have shown that the PVT modulates the hormonal and behavioral effects of both acute and chronic stress (Dong et al. 2020; Li et al. 2010; Pliota et al. 2018; Bhatnagar and Dallman 1998, 1999; Bhatnagar et al. 2003, 2002, 2000; Jaferi and Bhatnagar 2006; Jaferi et al. 2003; Heydendael et al. 2011). Homoeostatic challenges such as the physiological demands of thirst, hunger and disruptions in circadian rhythms also increase the activity of neurons in the PVT (Kirouac 2015; Colavito et al. 2015; Millan et al. 2017; Penzo and Gao 2021), suggesting that the PVT is activated by psychological and physiological stressors that have arousal effects on the CNS (reviewed in Hsu et al. 2014; Kirouac 2015). Accordingly, the PVT appears to be part of a neural network involved in coordinating a wide range of adaptive responses needed to maintain homeostasis (discussed in Li et al. 2021).

The source of brainstem afferents to the PVT has been the subject of a number of retrograde tracing studies. Early investigations with large injections of tracers in the PVT labeled neurons in a number of brainstem regions including the locus ceruleus (LC), nucleus of the solitary tract (NTS) and ventrolateral medulla (VLM) (Cornwall and Phillipson 1988; Chen and Su 1990). Studies with injection sites restricted to a single nucleus were generally in agreement that the PVT and other midline thalamic nuclei received afferents from a relatively small number of neurons in the LC, NTS and VLM (Krout et al. 2002, 2003; Krout and Loewy 2000a, b) some of which synthesize catecholamines (Phillipson and Bohn 1994; Otake and Ruggiero 1995; Otake et al. 1994). Brainstem catecholamine neurons regulate physiological and behavioral responses to homeostatic challenges via ramifying projections to many regions of the brain (Guyenet et al. 2013; Card et al. 2006; Rinaman 2011; Guyenet 2006; Dampney 1994; Aston-Jones and Cohen 2005). Recent papers have reported that catecholamine inputs to the PVT from the LC and VLM, respectively, modulate aversive stress and hypoglycemia-induced feeding responses (Beas et al. 2018; Sofia Beas et al. 2020). However, the existence of a functionally relevant catecholamine input to the PVT from the lower brainstem is not unequivocally supported by all anatomical studies. For instance, our group reported that injections of the retrograde tracer cholera toxin B (CTB) restricted to the PVT rarely produced labeling in the LC whereas the NTS and VLM contained only a few scattered neurons per brain section examined (Li and Kirouac 2012). In contrast, the parabrachial nucleus (PB) located around the superior cerebellar peduncle was consistently found to contain labeled neurons with the lateral aspect of this nucleus (LPB) containing more PVT projecting neurons than any other area of the brainstem (Li and Kirouac 2012; Kirouac et al. 2006; Li et al. 2014; Krout et al. 2002; Krout and Loewy 2000a). The PB is a multisensory integrating region primarily composed of glutamate neurons that have been shown to regulate a variety of behavioral responses associated with maintaining homeostasis including danger and distress (Chiang et al. 2019; Jaramillo et al. 2021; Palmiter 2018). For instance, the LPB is conceptualized as a major relay for viscerosensory and nociceptive signals to the forebrain and brainstem (Saper and Loewy 1980; Herbert et al. 1990; Moga et al. 1990; Palmiter 2018) where some of these projections have been shown to modulate arousal and behavioral responses to aversive conditions (Chiang et al. 2019; Jaramillo et al. 2021; Palmiter 2018). Afferent inputs to the LPB include catecholamine neurons in the NTS and VLM in addition to forebrain regions densely innervated by PB neurons (Tokita et al. 2009; Moga et al. 1990). Neurons in the PB project broadly to many areas of the forebrain and brainstem involved in maintaining homeostasis (Huang et al. 2021a; Saper and Loewy 1980).

These anatomical observations led us to question the potential importance of a direct projection to the PVT from catecholamine neurons in the lower brainstem and pointed to a more likely possibility that the LPB may serve as an integrating and relay center for afferents from catecholamine neurons to the PVT. To address these questions, we examined the distribution of labeled neurons in brainstem catecholamine cell groups following injections of CTB in anterior (aPVT) and posterior aspect of the PVT (pPVT). We also tested the hypothesis that the LPB is a relay center for brain inputs to the PVT by determining the source of input cells to LPB-PVT projecting neurons using a monosynaptic rabies tracing approach called tracing the input–output organization (TRIO) (Schwarz et al. 2015).

## Methods

### Animals

A total of 34 male Sprague–Dawley rats (University of Manitoba vivarium) were used to complete the experiments described in the paper. The rats weighed approximately 300 ± 10 g at the time of the injections and were housed on a 12:12 h light–dark cycle with food and water freely available. All experiments were carried out according to guidelines of the Canadian Council on Animal Care and approved by Research Ethics Review Board of the University of Manitoba.

### Injections of CTB for retrograde tracing experiments

Rats were anesthetized with 2–3% isoflurane and given meloxicam (2 mg/kg, s.c.) for post-surgical pain management. The animals were placed in a Stoelting stereotaxic frame and a hand drill was used to expose the brain surface above the midline thalamus. Dual injections of CTB were made using glass pipettes (approximately 40 µm diameter) directed at the aPVT (1.8 mm posterior, 1.0 mm lateral, 5.4 mm ventral, 10° angle toward the center; all coordinates are relative to bregma and bone surface) and the pPVT (3.1 mm posterior, 1.0 mm lateral, 5.3 mm ventral, 10° angle toward the center) on the right side of the brain of the same animal. The CTB injected was conjugated to Alexa Fluor-488 (AF-488-CTB, C22841, Invitrogen, Carlsbad, CA, USA) or Alexa Fluor-594 (AF-594-CTB; C22842, Invitrogen) with one of these conjugates given in either the aPVT or pPVT of the same animal. The CTB solids were dissolved at a 0.5% concentration in 0.06 M neutral phosphate buffer and 250 nl of the solution was administered over a 15 min period using a pressure injection device (Picospritzer, Park Hannifin, Hollis, NH, USA). The scalp was sutured and rats returned to their home cages for approximate 10 days prior to being deeply anesthetized with 10% chloral hydrate (600 mg/kg, i.p.) and transcardially perfused with 150 ml heparinized saline followed by 400–500 ml ice-cold 4% paraformaldehyde in 0.1 M phosphate buffer (pH 7.4). The brains were removed and post-fixed in the same fixative overnight and cryoprotected in phosphate buffered saline (PBS) containing 20% sucrose and 10% glycerin at 4 °C for 48 h. Coronal sections of the brain were taken at 50 µm with a cryostat (UltroPro 5000) and stored in cryoprotectant before the immunoreactions. Finally, a few rats (*n* = 3) were perfused and brains post-fixed as above and sections of the thalamus were obtained to carry out immunoreactions against tyrosine hydroxylase (TH).

### Injections of viral agents for TRIO experiments

**Fig. 1:**
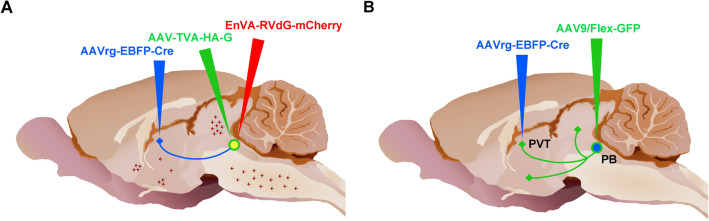
Approaches used for microinjection of viral vectors. **a** Injections for the TRIO experiments. **b** Injections for the intersectional anterograde tracing experiments. Adapted from sagittal brain images created by Gill Brown, King’s College, London. See list for abbreviations.

Microinjections of viral preparations were done in two surgical procedures using similar methods as the CTB experiments (Fig. 1A). In the first procedure, rats received 350 nl injections of an adeno-associated virus (AAV) that expresses the Cre-recombinase transgene in the retrograde direction (AAVrg-Syn1-EBFP-Cre; 7.6 × 10^12^ copies/ml; #51,507-AAVrg, Addgene, Cambridge, MA, USA) in both the aPVT and pPVT using the following coordinates: aPVT (1.8 mm posterior, 1.0 mm lateral, 5.9 mm ventral, 10° angle toward the center) and the pPVT (3.1 mm posterior, 1.0 mm lateral, 5.8 mm ventral, 10° angle toward the center) in addition to a 500 nl injection of a Cre-dependent “helper AAV” (AAV2/1 hSyn-Flex-TVA-HA-G, 1.0 × 10^11^ copies/ml; NTNU Viral Vector Core, Kavli Institute, Norway) in the LPB according to the following coordinates: 7.5 mm posterior, 1.8 mm lateral, 7.0 mm ventral, 14° in the anterior to posterior direction. The helper AAV transduces the expression of TVA and a component of the rabies glycoprotein in LPB-PVT neurons that contain the Cre from injections of the AAVrg-EBFP-Cre in the PVT. The avian receptor TVA promotes infection with a rabies virus pseudotyped with the envelop protein from avian sarcoma leucosis virus type A (EnvA) and the glycoprotein (G) provides the G-deleted rabies virus the critical component for transsysnaptically infecting input cells (Lavin et al. 2019; Wickersham et al. 2007). After 2 weeks, an injection of 500 nl of G-deleted-SADB19G-EnvA-Rabies-mCherry (RVdG-mCherry, 1.0 × 10^10^ copies/ml; NTNU Viral Vector Core) was made in the LPB using the same coordinates as for the helper AAV. Control experiments were done by excluding either the AAVrg-EBFP-Cre injections in the PVT (*n* = 3) or the helper AAV injection in the LPB (*n* = 3) prior to the RVdG-mCherry injection in the LPB. Rats were perfused with fixative 7 days after the injection of the RVdG-mCherry and the brains collected for sectioning as described for the CTB experiments.

### Injections of viral agents for intersectional anterograde tracing experiments

The approach involves transducing Cre-recombinase in a subpopulation of projection-specific neurons using an AAV that transports in the retrograde direction (AAVrg-EBFP-Cre) in combination with an AAV that transduces neurons and fibers in the anterograde direction (Fig. 1B). This results in the tracing of axonal fiber collaterals to different regions of the brain in addition to the area initially targeted by the AAVrg-EBFP-Cre (Li et al. 2021). Rats were prepared as described above and microinjections of solutions containing an AAVrg-Syn1-EBFP-Cre preparation (500 nl) were made in the right aPVT and pPVT of the same animal followed by a microinjection of an AAV9/Flex-GFP preparation (500 nl; 5.21 × 10^13^ GC/ml, Salk Institute Viral Vector Core, La Jolla, CA, USA) in the LPB on the same side of the brain. The coordinates for the aPVT, pPVT and LPB were the same as the TRIO experiments and animals were allowed a 4-week survival period before perfusion and the removal of the brain for sectioning.

### Tissue preparation

Immunoreactions for tyrosine hydroxylase were done on brainstem sections collected from both the CTB and the TRIO experiments. Sections of the LPB region were immunoreacted for human hemagglutinin (HA) to visualize the neurons transduced by the helper virus in the TRIO experiments. Finally, every sixth section was reacted for neuronal nuclear protein (NeuN) in some cases for each group of experiments to help establish the neuronal labeling based on cytoarchitectural boundaries of nuclei. All reactions were done in free-floating sections at room temperature and involved placing sections in a blocking solution of PBS containing 5% donkey serum, 0.3% of Triton X-100, and 0.1% of sodium azide for 1 h prior to incubation in the antiserum. The primary and secondary antibodies were diluted in the blocking solution and sections were rinsed in PBS between each step. The immunoreactions were done as described below followed by a final set of rinses before mounting and placing a coverslip using Fluromount-G (Southern Biotech, Birmingham, AL, USA).

#### Tyrosine hydroxylase (TH)

Sections of the brainstem that contained CTB labeled neurons or mCherry neurons were incubated in a solution containing a mouse anti-TH monoclonal antibody (1:10,000; T-2928, Sigma-Aldrich, Oakville, ON, Canada) for 2 days. After several rinses, sections were transferred to a secondary antibody solution containing Alexa-Fluor Plus 647 (1:3000, A32787, Invitrogen) for CTB sections and Alexa-Fluor Plus 488 (1:3000, A32766, Invitrogen) for mCherry sections for 2 h. Sections of PVT were incubated in the same TH antibody solutions before transferred to a secondary Alexa-Fluor Plus 594 donkey anti-mouse antibody (1:3000; A32744, Invitrogen) for 2 h.

#### Human hemagglutinin (HA)

After one hour in the blocking solution, every sixth section of the LPB was incubated in a primary antibody solution containing rabbit anti HA-Tag (C29F4) monoclonal antibody (1:1000; 3724 s, Cell Signaling Technologies, Danvers, MA, USA) overnight followed by incubation in a solution containing a secondary Alexa-Fluor Plus 488 donkey anti-rabbit antibody (1:3000; A32790, Invitrogen) for 2 h.

#### Neuronal nuclear protein (NeuN)

Sections were incubated in a primary mouse anti-NeuN antibody (1:1000; MAB377, Chemicon, Temecula, CA, USA) overnight followed by incubation in a solution containing Alexa-Fluor Plus 488 donkey anti-mouse (1:3000) for 2 h.

### Image and quantitative analysis

Images showing the distribution of neurons and fibers were produced from compiled stacks of frames captured using Zeiss Axio Observer Z1 microscope equipped with Axiocam 503 mono camera. The frames were taken with 10x objective lens with X and Y and Z stacks (2 µm increments) set to cover a region of interest. The exposure time was adjusted for each individual channel to optimize the images captured and kept consistent for each case in the different experiments. Images were processed using “Stitch” to fuse the X and Y tiles and then “Extended Depth of Focus” for the Z stacks (Zen Blue, Zeiss) to produce the tiled images. Mapping and quantification of labeled neurons in the various experiments were done using a standardized gamma setting for each color channel. The contrast of the composite images was adjusted in Adobe Photoshop to produce the final images for the figures. Cell counts and mapping of the labeled neurons were done according to methods described below.

#### Retrograde CTB tracing experiments

Single-labeled neurons were identified and marked on images viewed in Zen Blue with the appropriate color channel whereas multi-labeled neurons were identified in images with merged color channels. The soundness of single-cell multi-labeling was further evaluated by switching between color channels to confirm a clear overlap of signals within the same soma. Images with marked single- and multi-labeled neurons were imported to Adobe Illustrator CS4 and overlaid with an image file of the appropriate anatomical level of the brain from the digital atlas of the rat brain (Paxinos and Watson 2009). The boundaries of brain nuclei and fiber bundles from the atlas images were adjusted slightly to correspond to the microscopic image. The merged image was exported to Adobe Photoshop and the locations of the labeled neurons in the brain were generated by manually placing a distinctive symbol to indicate the location of a labeled neuron. The final image files of individual stereotaxic levels were used to generate the figures displaying labeled neurons on selected stereotaxic levels of the brain.

#### TRIO experiments

The number of starter cells near the superior cerebellar peduncle and input cells for the entire brain were quantified on coronal sections captured at 300 µm intervals. Starter cells were identified as neurons with unambiguous co-distribution of mCherry and HA within the same neuronal cell body using the same methods as the CTB experiments. The number of RVdG-mCherry input cells for the different regions of the brain was quantified for those regions that had 50 or more input cells over the series of sections. The mCherry labeled cells were marked using a circular symbol on the captured images in Photoshop and the number of marked cells was quantified using ImageJ (Fuji).

#### Intersectional anterograde tracing experiments

The number of GFP labeled neurons in the PB was quantified by counting the single-labeled GFP neurons from sections spanning the nucleus. The density and location of fiber labeling are described qualitatively and the figures showing the labeling were produced from images captured as described above.

### Statistical analyses

The data for the CTB tracing experiments were analyzed using one-way ANOVA followed by Bonferroni multiple comparison tests using the statistical software OriginPro 8 (OriginLab Corporation, Northampton, MA, USA). An adjusted value of *p* < 0.05 was considered to be significant and the data are presented as mean ± SEM.

## Results

### CTB retrograde tracing

**Fig. 2:**
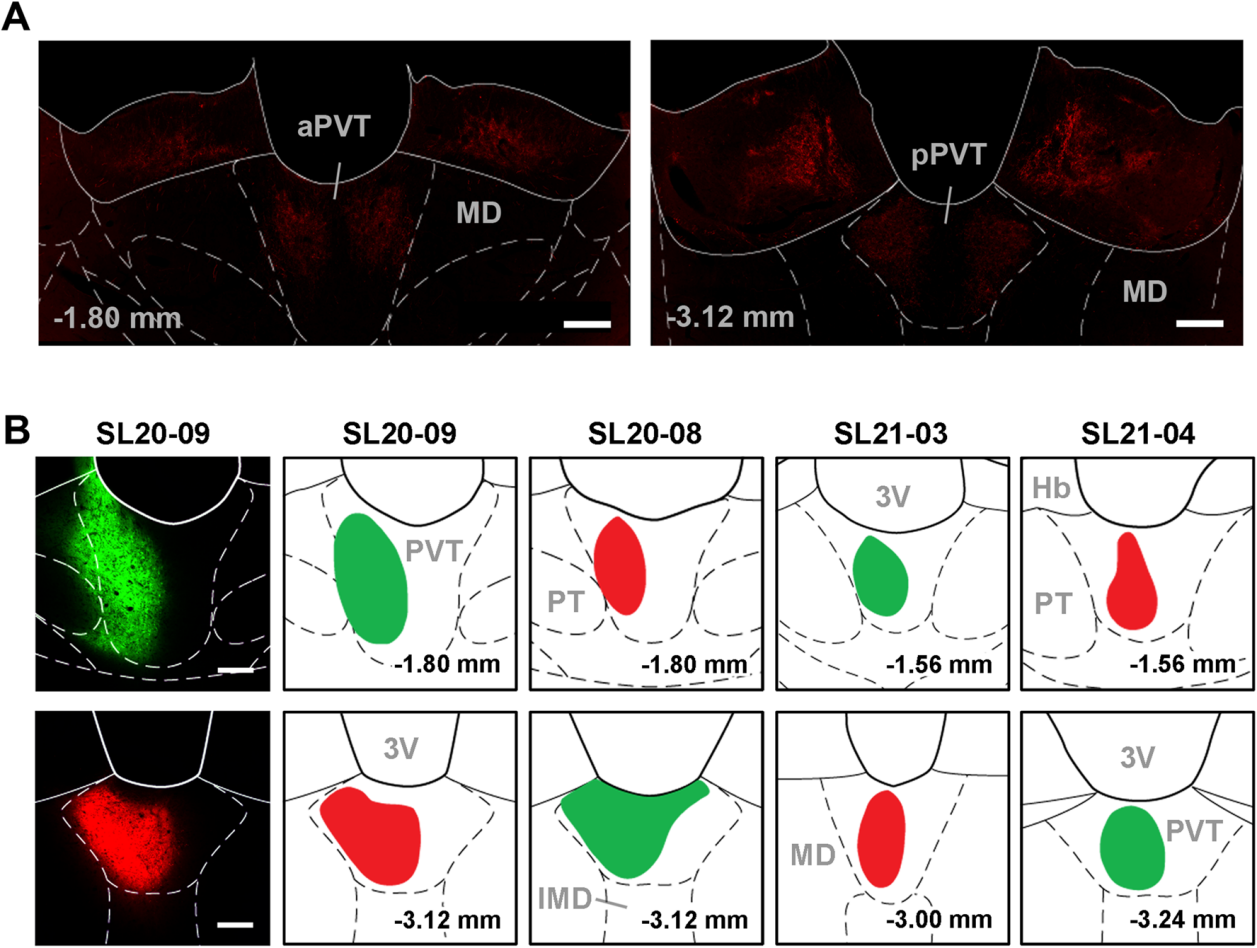
Tyrosine hydroxylase (TH) fiber labeling in the PVT and the location of the CTB injections. **a** Immunofluorescence for TH is comparatively weaker in the aPVT (left) and pPVT (right). **b** Location of combination injections of AF-488-CTB (green) and AF-594-CTB (red) in the aPVT (upper row) and pPVT (lower row) in 4 cases (columns) used for the retrograde tracing analysis. See list for abbreviations. Numbers at the bottom represent distance from the bregma. Scale bars: 200 µm

**Fig. 3:**
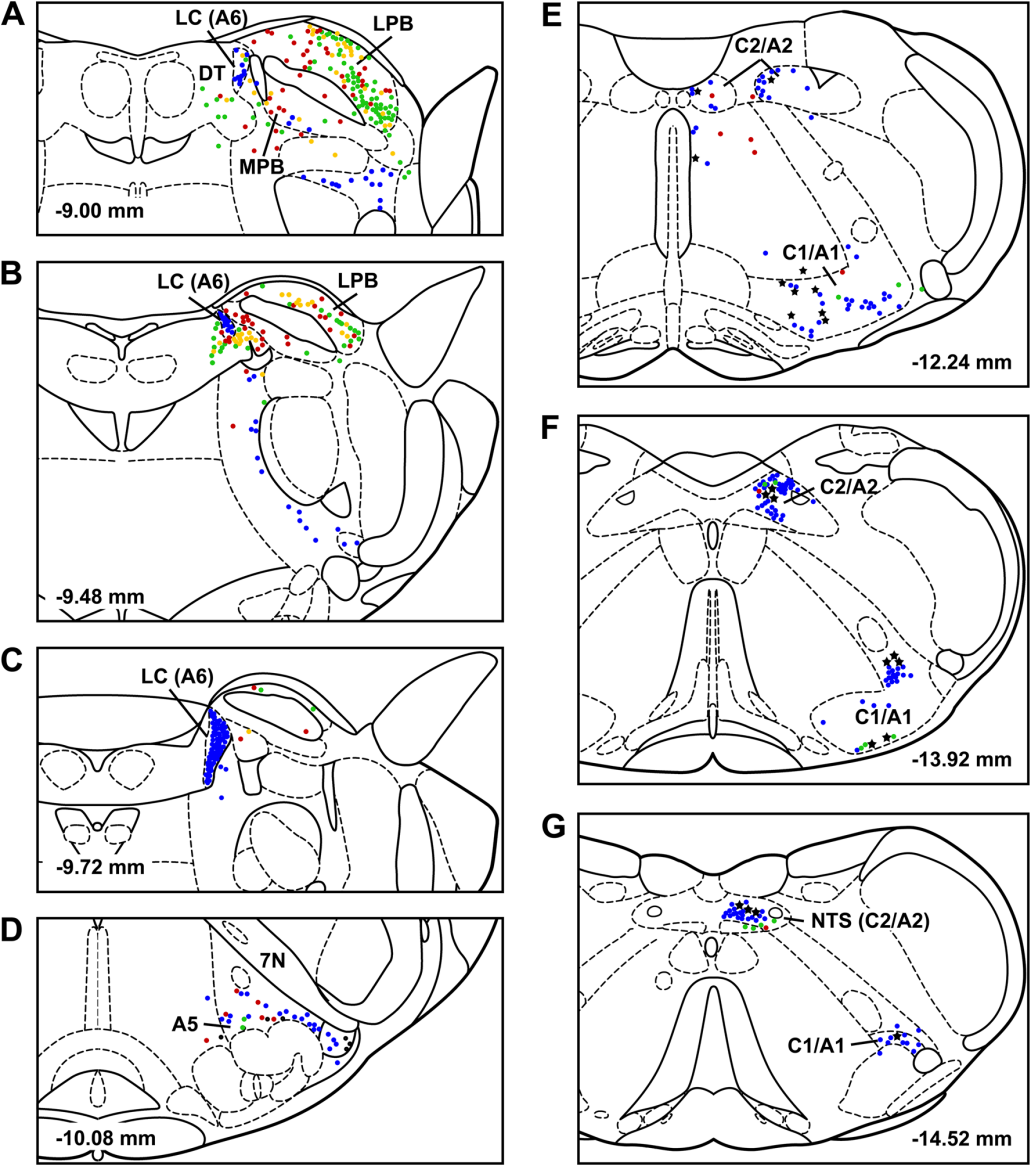
Location of labeled cells in the lower brainstem following injections of CTB in the PVT. Single CTB labeled neurons from injections in the aPVT (green) and pPVT (red) and cells projecting to both the aPVT and pPVT (yellow) are shown along with the location of TH positive cells (blue) as well as cells labeled with both CTB from either the aPVT or pPVT and TH (black stars) for case SL20-09 plotted onto a series of drawings of a rat stereotaxic atlas (Paxinos and Watson 2009) extending from the pons to the caudal medulla (**a**–**g**). See list for abbreviations and the numbers at the bottom represent distance from the bregma

**Fig. 4:**
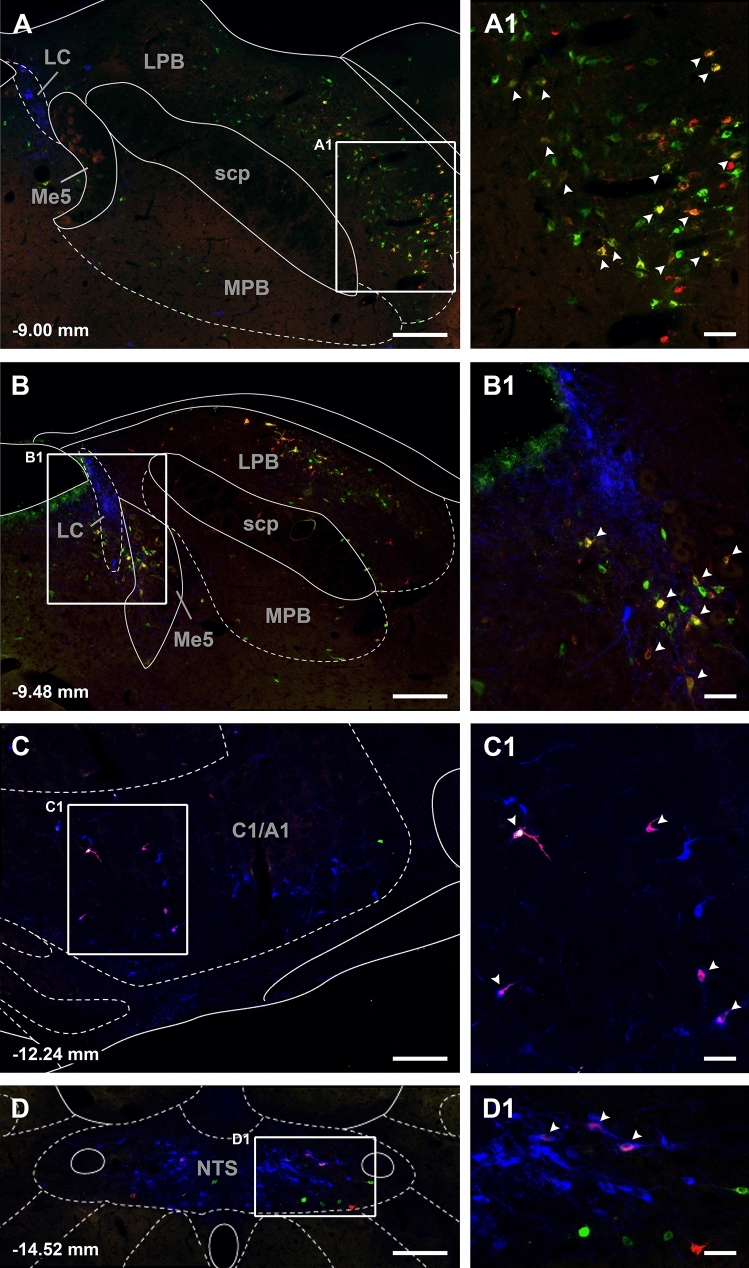
Images of CTB labeled neurons in the pons and medulla. Single CTB labeled neurons from injections in the aPVT (red) and pPVT (green) in addition to cells projecting to both the aPVT and pPVT (yellow-orange, arrow heads in **a1** and **b1**) are visible for case SL20-08. Also visible are single-labeled TH positive cells (blue) and double labeled TH and CTB cells (cyan) as indicated by arrow heads in the numbered insets (**c1** and **d1**). The images are arranged from anterior to posterior levels (**a**–**d**) with the numbers at the bottom representing distance from the bregma. See list for abbreviations. Scale bars: 200 µm for the images, 50 µm for the insets

The aPVT and pPVT contain a weak plexus of TH fibers (Fig. 2A). Retrograde tracing experiments were done to further evaluate if catecholamine neurons in the lower brainstem are the source of these fibers. Four cases with combination injections of CTB restricted to the aPVT and pPVT were used to generate the figures and data analysis (Fig. 2B). Table 1 displays the results of the analysis of the number of CTB neurons in the lower brainstem. The LPB contained the largest number of neurons whereas the medial PB (MPB) contained fewer neurons (Figs. 3A, B; 4A, B). The catecholamine cell groups of the lower brainstem contained a relatively small number of retrograde labeled neurons (Figs. 3, 4). Table 2 displays the number of CTB labeled neurons that were TH positive indicating that catecholamine neurons represent a small proportion of all the neurons in the pons and medulla that project to the PVT. Indeed, a few neurons per section were observed in the catecholamine cell groups embedded in the VLM (Figs. 3E–G, 4C) and the NTS (Figs. 3E–G, 4D). The LC was devoid of double labeled neurons in all of the sections of the LC examined (Figs. 3A–C; 4A, B). However, these latter findings should be interpreted based on an understanding that the LC is a small compact nucleus composed of fewer neurons than the LPB. In summary, the CTB tracing results support our previous observations that brainstem neurons innervating the PVT originate primarily from the LPB and not from the LC, NTS, VLM (Li and Kirouac 2012; Kirouac et al. 2006).

**Table 1:** Number of retrograde CTB cells according to brainstem regions

Region	aPVT	pPVT	Double
LPB	202.5 ± 49.3^a^	224.25 ± 65.9^a^	69.75 ± 16.3^a^
A6	21.5 ± 3.6	41 ± 11.5	6.75 ± 1.0
A5 (rVLM)	1 ± 1	2.5 ± 0.9	0
C1/A1 (cVLM)	58.25 ± 19.1	64.5 ± 11.4	9.75 ± 3.8
C2/A2 (NTS)	63.25 ± 8.6	66 ± 9.8	13.25 ± 1.9

aPVT F (4,15) = 10.78, *p* = 2.55E−04; pPVT F (4,15) = 7.65, *p* = 1.45E−03; double F (4,15) = 16.10, *p* = 8.77E−09

^a^Significantly different compared to A6, rVLM, cVLM and NTS

**Table 2:** Number of CTB and TH positive cells in the brainstem

Region	aPVT	pPVT	Double
C1/A1 (cVLM)	22.5 ± 6.0^a^	29.25 ± 8.5^a^	15.5 ± 5.0^a^
C2/A2 (NTS)	12.5 ± 1.9	22.5 ± 6.7	7.5 ± 2.4
A6	0.25 ± 0.25	0.25 ± 0.25	0.25 ± 0.25
A5 (rVLM)	0	0	0

aPVT F (3,12) = 11.71, *p* = 7.06E−04; pPVT F (3,12) = 7.78, *p* = 3.78E−03; double (3, 12) = 7.04, *p* = 5.5E−03

^a^Significantly different compared to A6 and rVLM; double refers to CTB+/TH+ cells from the aPVT and pPVT

### TRIO experiments

**Fig. 5:**
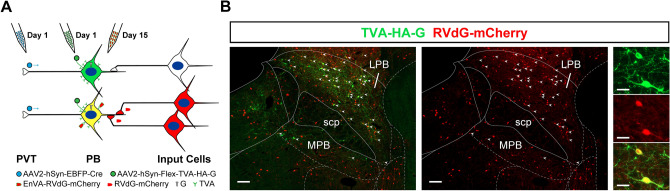
Schematic of the TRIO approach (**a**) and resulting starter cells in PB neurons that project to the PVT (**b**). The presence of starter cells is indicated by small white arrowheads at low magnification (left side) and examples are shown at higher magnification (right side) in **b**. See list for abbreviations. Scale bars: 50 µm for the lower magnification images and 10 µm for the higher ones

**Fig. 6:**
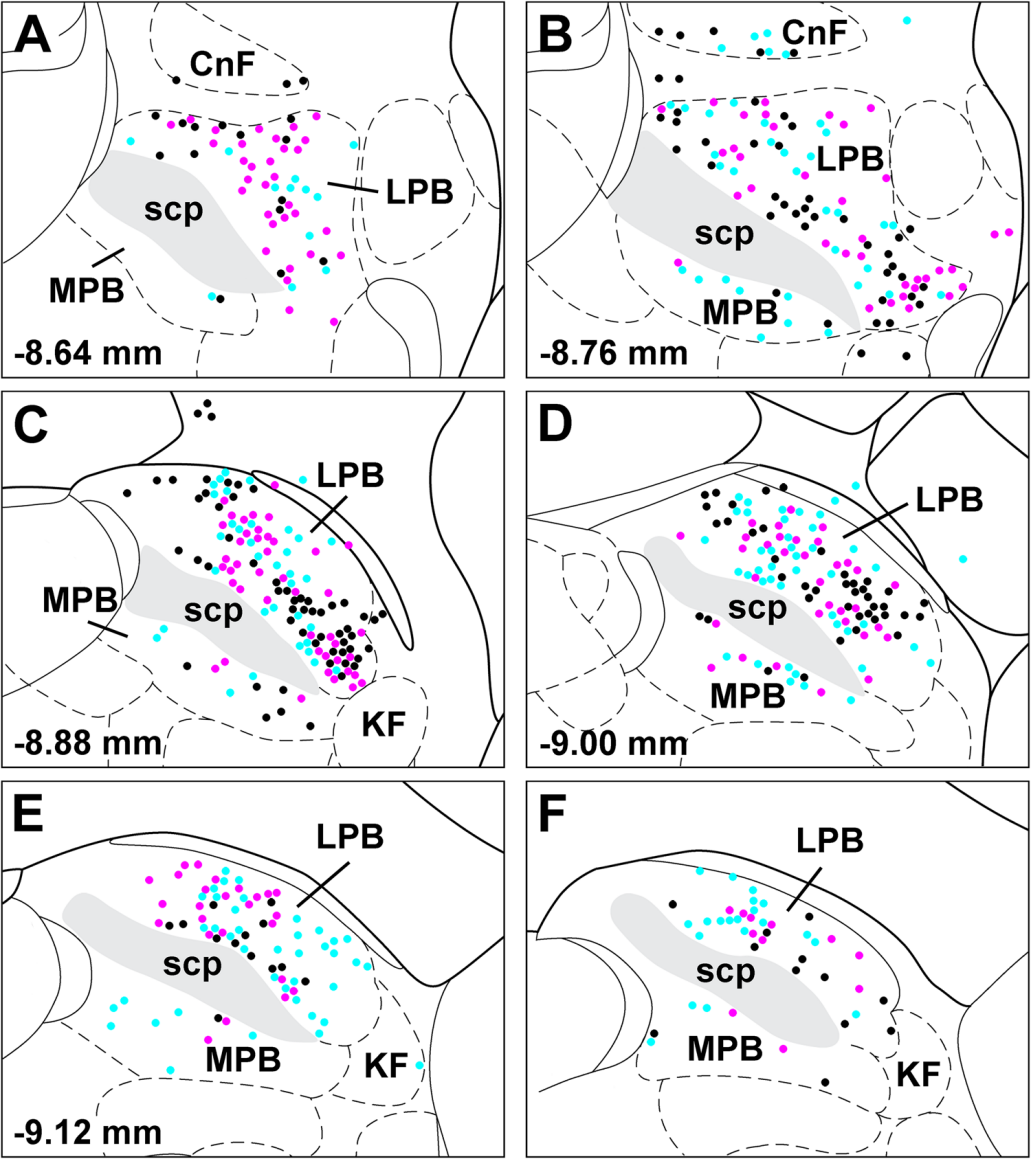
Location of starter cells in the cases used for quantitative analysis of input cells to the LPB-PVT neurons. The location of the starter cells derived from each case (*n* = 3) is uniquely color-coded and is shown from anterior to posterior levels of the PB (**a**–**f**). See list for abbreviations. Numbers at the bottom represent distance from the bregma

Starter cells were identified by the presence of mCherry and HA in the same PB neuron (Fig. 5). The number of input cell per PB-PVT starter cell was quantified for the whole brain in three cases where the AAVrg-Cre injections were limited to the aPVT and pPVT based on the presence of the reporter EBFP (see intersectional anterograde tracing results for examples of EBFP expression at the PVT injection site). Starter cells were located primarily in the LPB (Figs. 5B, 6) in a pattern that is similar to what was observed in the CTB tracing experiments. A small number of starter cells were located in the MPB and anterodorsally in the transition area between the LPB and the nucleus cuneiformis. The number of starter cells was consistent for the 3 cases examined (totals of 221, 209, and 205 starter cells in the PB sections counted for the 3 cases).

**Fig. 7:**
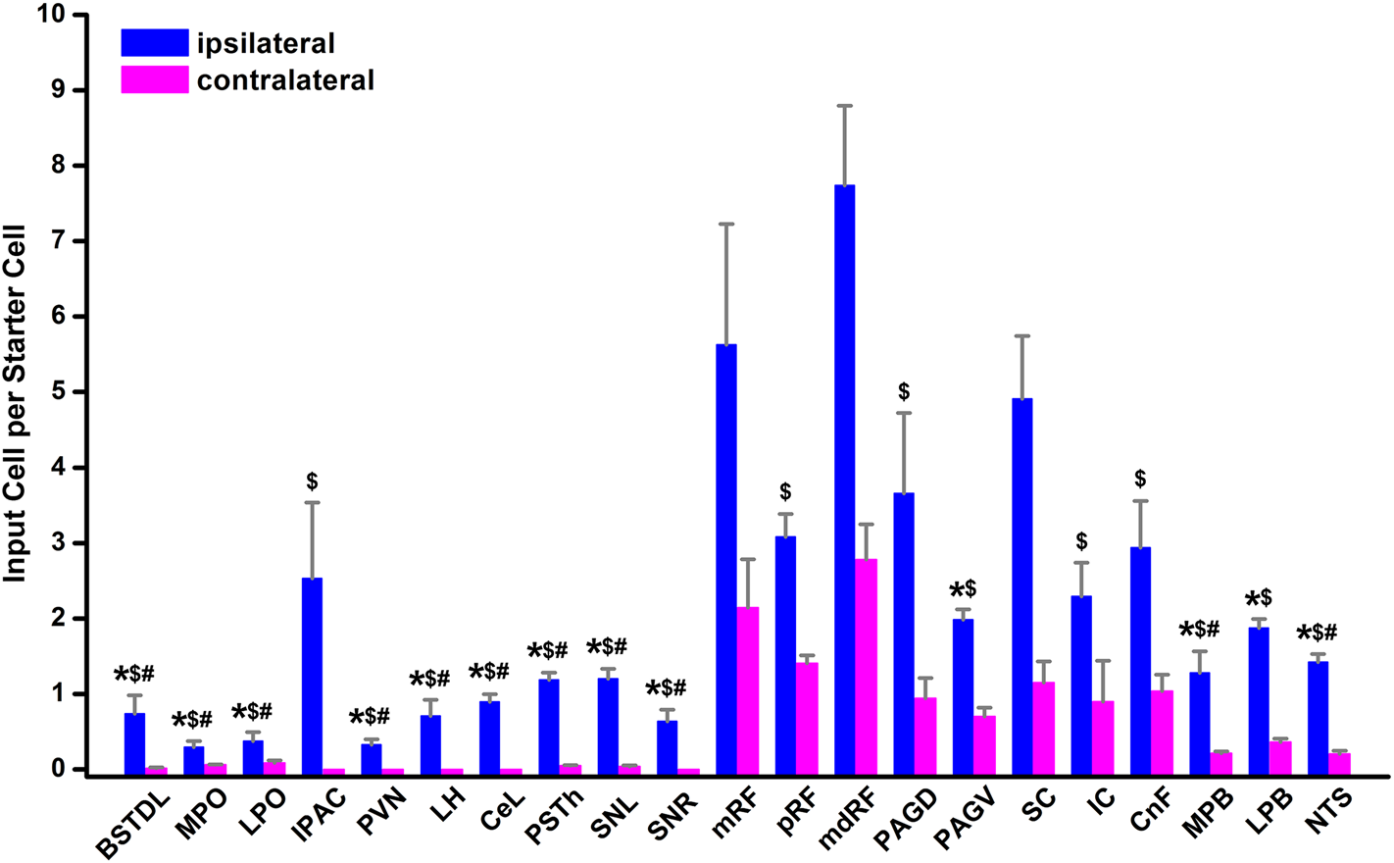
Number of input cell per starter cell from different regions of the brain making synapses on LPB-PVT projecting neurons. The brain regions quantified are listed from anterior to posterior levels. See list for abbreviations. *significantly different from mRF, ^$^significantly different from mdRF, ^**#**^significantly different from SC; *p* < 0.05

**Fig. 8:**
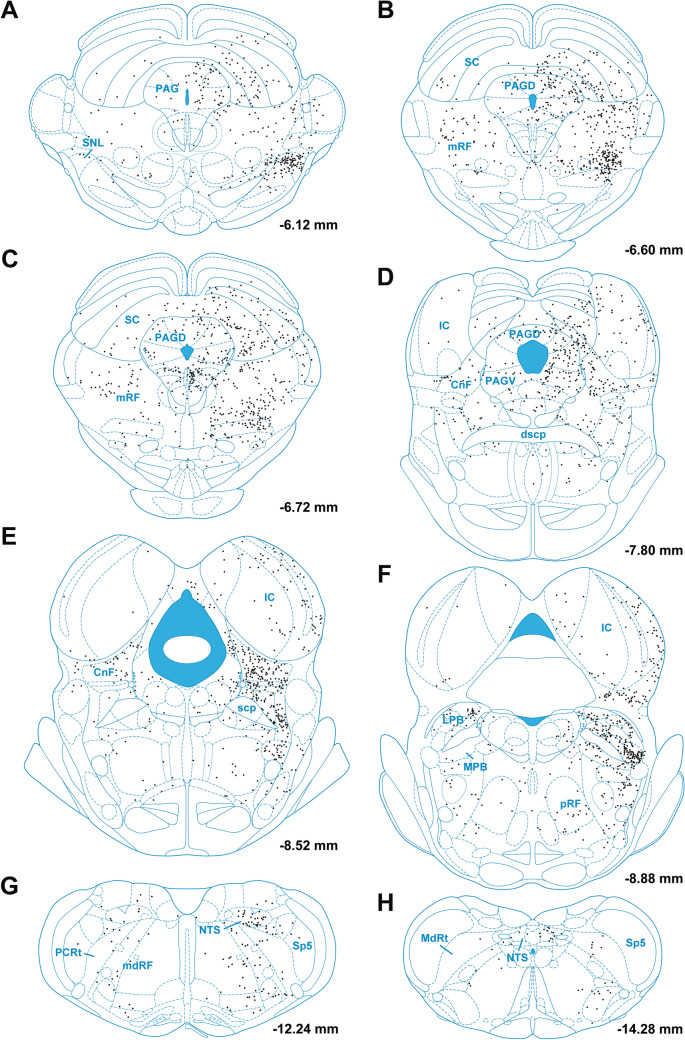
Drawings showing the location of brainstem cells providing monosynaptic input to LPB-PVT neurons in one representative case (case with starter cells indicated as magenta colored symbols in Fig. 6). The locations of the input cells are shown from anterior to posterior levels of the brainstem (**a**–**h**). See list for abbreviations and numbers at the bottom represent distance from the bregma

**Fig. 9:**
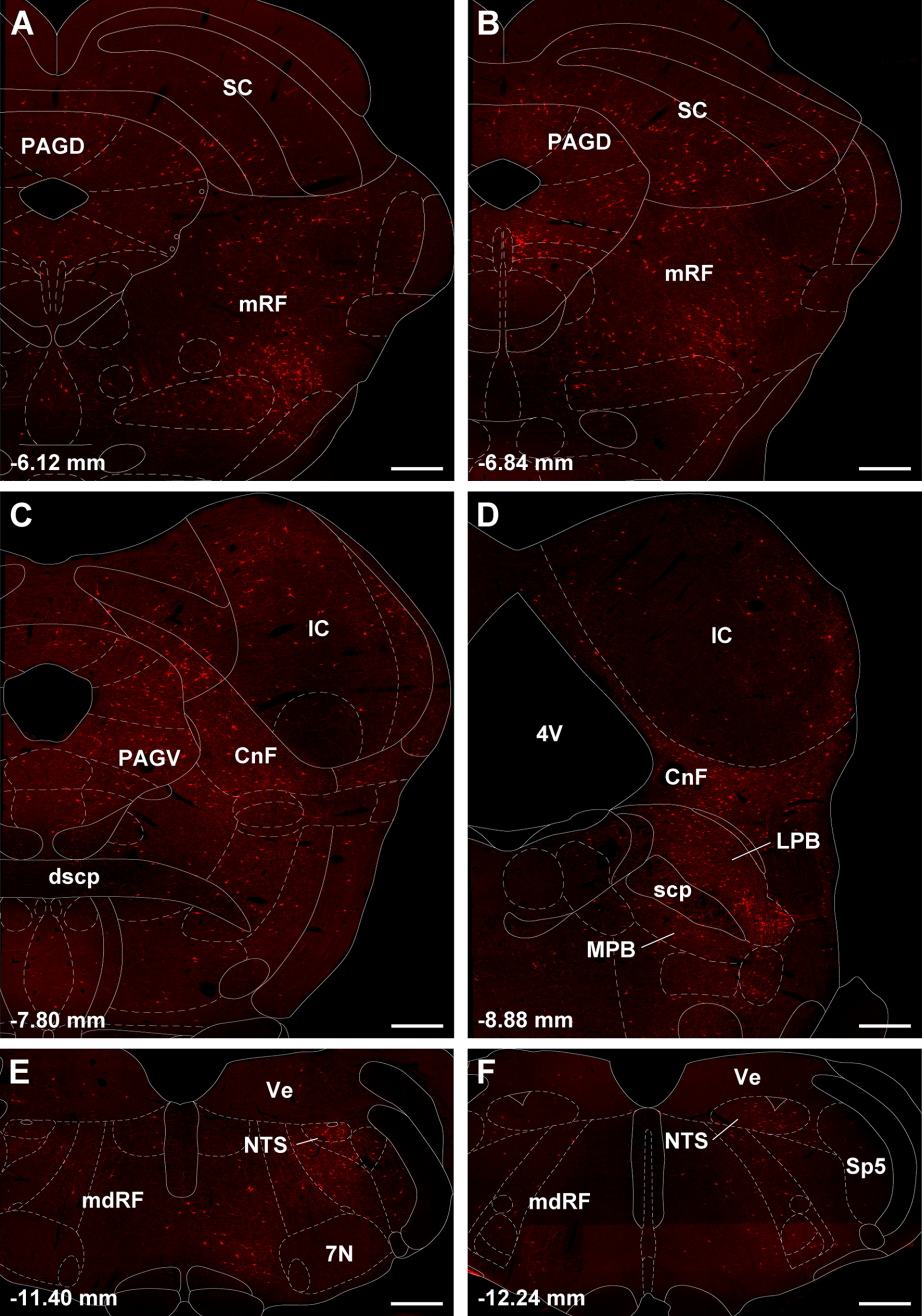
Images of brainstem sections showing mCherry labeling from the TRIO approach applied to LPB-PVT neurons in the case shown in Fig. 8. The sections are shown from the anterior to posterior levels of the brainstem (**a**–**f**). See list for abbreviations and numbers at the bottom represent distance from the bregma. Scale bars: 500 µm

Figure 7 shows the number of input cell per starter cell from regions of the brain that consistently contained input cells in the 3 cases quantified. Input cells were found bilaterally with the cells located on the same side of the injection being more numerous. There was a significant difference in the number of the input cells provided in the different regional groups quantified (F_(20,42)_ = 10.63, *p* < 0.0001) with the reticular formation (RF) containing the largest number of input cells (Fig. 7). Input cells were broadly scattered in the RF of the midbrain (Figs. 8A–D; 9A–C), pons (Figs. 8E, F, 9D) and medulla (Figs. 8G, H; 9E, F). The lateral substantia nigra including its transition into the retrorubral field embeded in the midbrain RF was a consistent source of input cells (Figs. 8A–C; 9A, B). These input cells were not immunopositive for TH despite being intermixed with dopaminergic neurons (data not shown). Subregions of the periaqueductal gray (PAG) including the dorsal, lateral and ventrolateral regions were a regular source of input cells to LPB-PVT neurons (Figs. 8A–D; 9A–C). Both the superior and inferior colliculi of the tectum provided a substantial number of input cells (Figs. 8A–F; 9A–D). The nucleus cuneiformis (CnF) and various regions of the PB contained input cells that would appear to make short intra-nuclear axonal connections (Fig. 8E, F; 9C, D). Input cells were scattered in the NTS and VLM over a number of stereotaxic levels (Figs. 8G, H; 9E, F), most of which were immunonegative for TH (data not shown). A notable collection of input cells was observed in the anterior NTS and the adjacent parvocellular reticular nucleus in the medulla, a distinctive nucleus of the medullary RF (Figs. 8G; 9E).

**Fig. 10:**
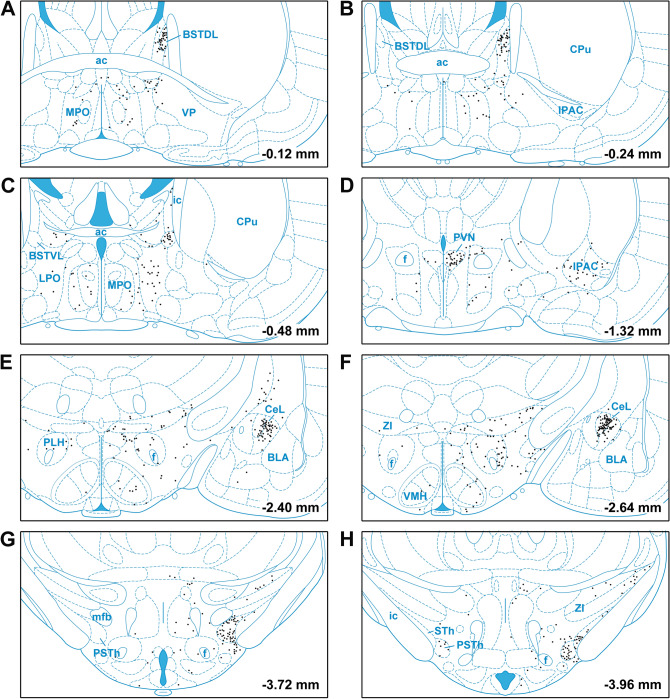
Drawings showing the location of the forebrain cells providing monosynaptic input to LPB-PVT neurons. The locations of the input cells are shown from the anterior to posterior most levels of the forebrain (**a**–**h**). See list for abbreviations and numbers at the bottom represent distance from the bregma

**Fig. 11:**
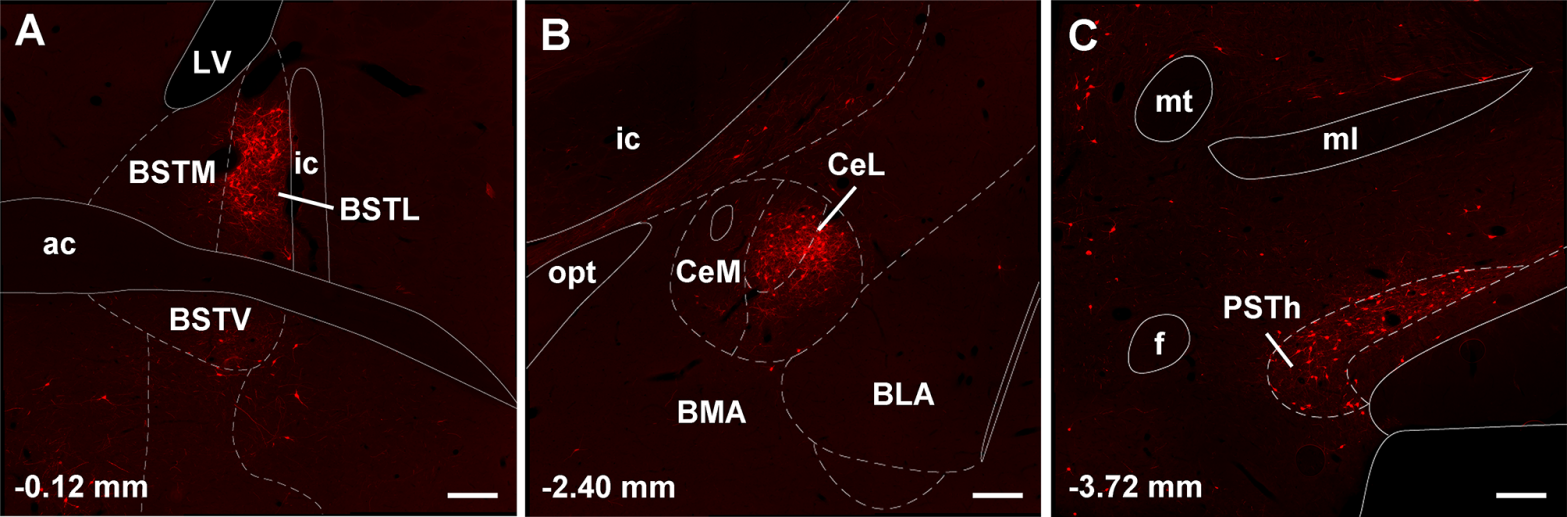
Images of the forebrain showing clusters of mCherry input cells making synapses on LPB-PVT projecting neurons. The sections are from the case shown in Fig. 10 and are arranged from the anterior to posterior most levels of the forebrain (**a**–**c**). See list for abbreviations and numbers at the bottom represent distance from the bregma. Scale bars: 250 µm

The forebrain provided fewer input cells to LPB-PVT neurons. However, in some instances, the input cells formed tight and distinctive clusters. Especially noteworthy were the clusters observed in the BSTDL (Figs. 10A, B; 11A), CeL (Figs. 10E, F; 11B), and parasubthalamic nucleus (Figs. 10G, H; 11C). Input cells were also found in the paraventricular nucleus of the hypothalamus (Fig. 10D), perifornical region of the lateral hypothalamus (Fig. 10E, F), and the interstitial nucleus of the posterior limb of the anterior commissure (Fig. 10D). Other regions of the forebrain only contained an occasional scattering of input cells and most regions including all of the cortex and striatum were entirely devoid of cells.

#### Control experiments

**Fig. 12:**
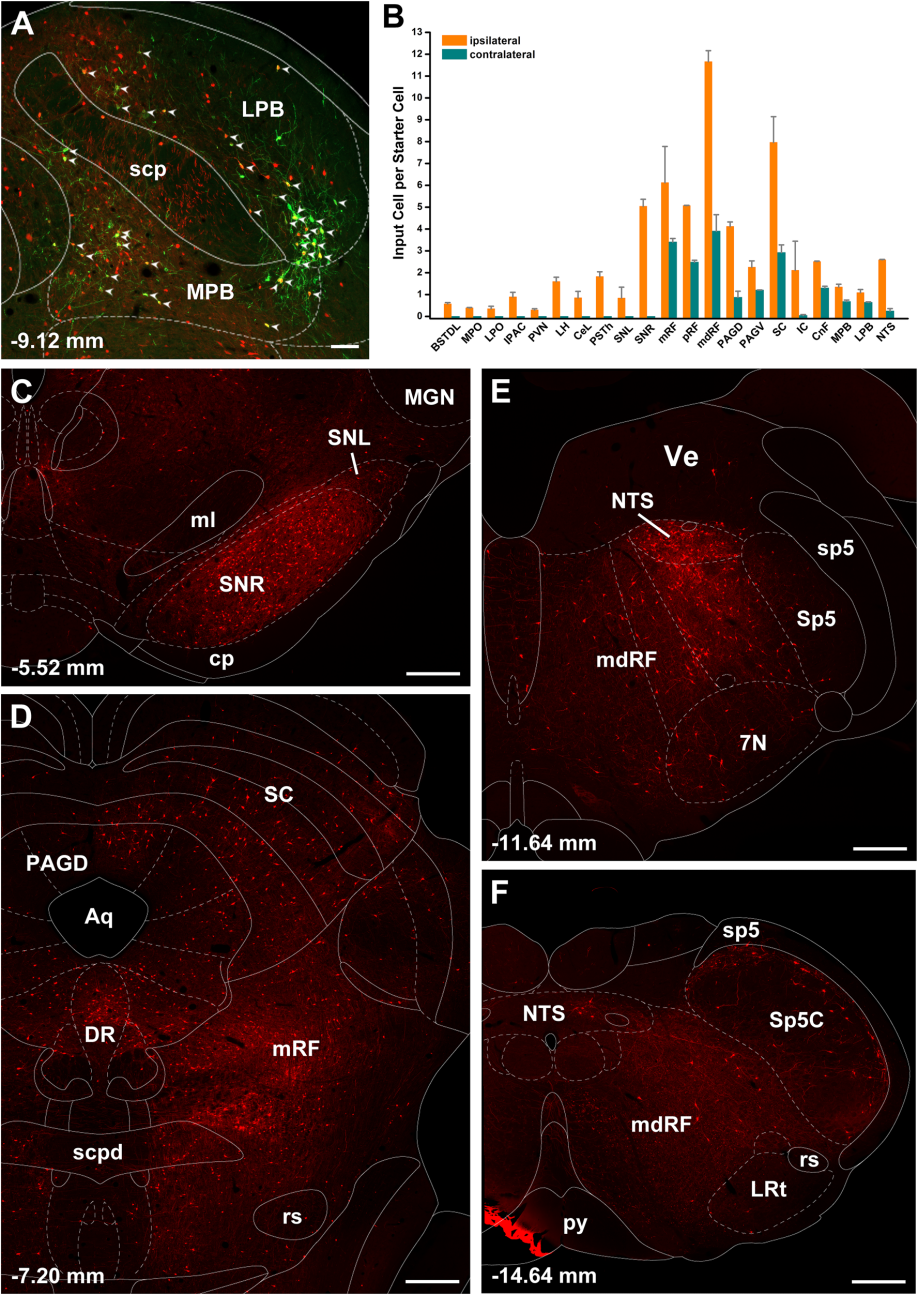
Input cells to the PB-CeL neurons. Image of the PB showing starter cells in one case (**a**) and the number of input cells per starter cells in the brain (**b**). Images of the brainstem showing mCherry input cells to PB-CeL projecting neurons arranged from the anterior to posterior most levels of the forebrain (**c**–**f**). See list for abbreviations and numbers at the bottom represent distance from the bregma. Scale bars: 100 µm for **a**, 500 µm for **c**–**f**

**Fig. 13:**
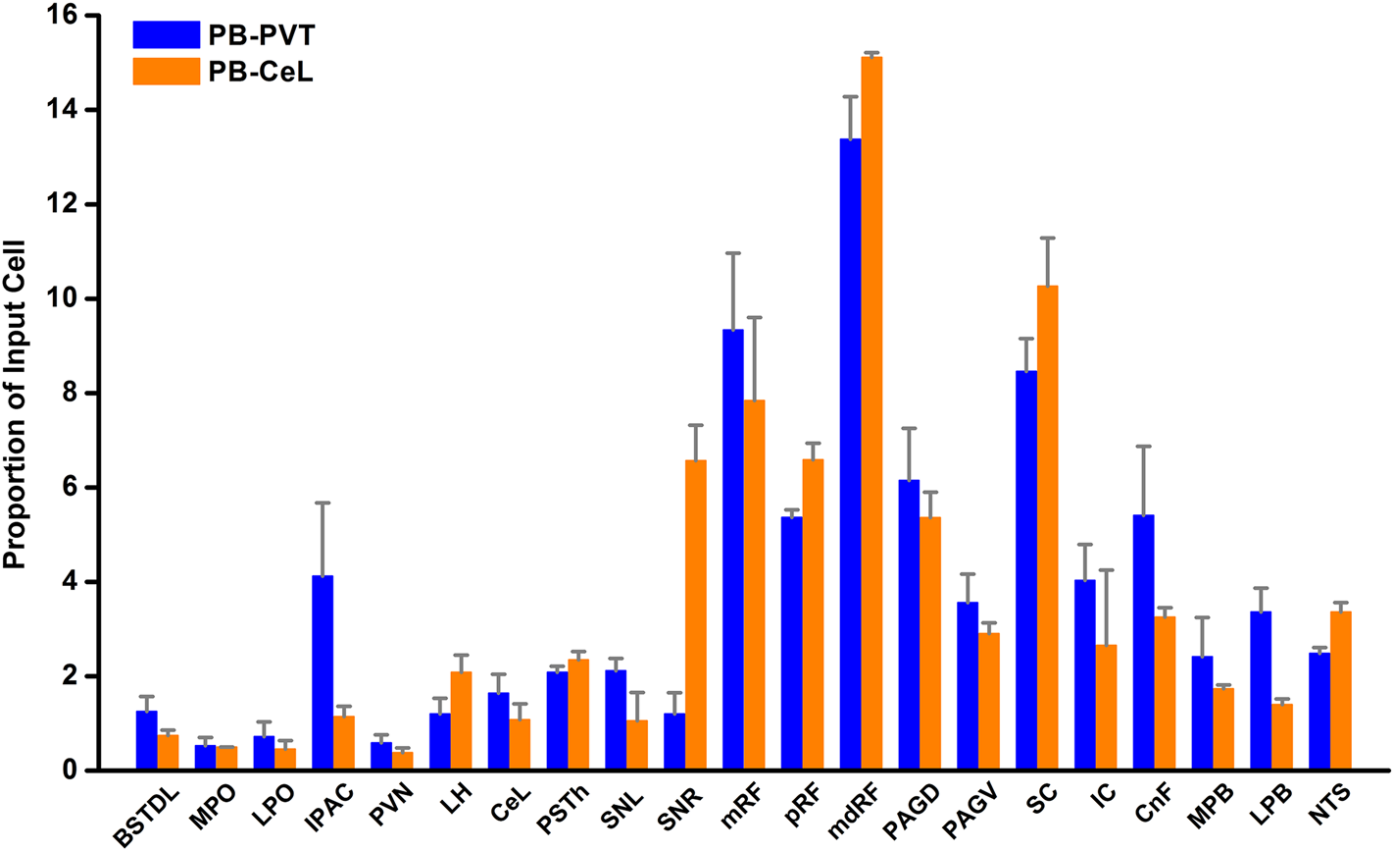
Proportion of input cells to PB neurons that synapse on the PVT or CeL

Starter cells for the experiments involving injections of AAVrg-EBFP-Cre in the CeL (*n* = 2, 302 and 331 starter cells) were located in the LPB and MPB following injections of the helper AAV and the RVdG in the LPB (Fig. 12A). There were more starter cells in the MPB associated with the CeL than with the PVT experiments. Input cells for PB-CeL experiments (Fig. 12B) were in similar brain regions as for the PB-PVT experiments. One notable exception was a larger number of input cells in the reticular portion of the substantia nigra in the PB-CeL cases (Fig. 12C), which was absent in the PB-PVT cases (Fig. 8A). Figure 13 shows the proportion of input cells from different regions of the brain associated with the PB-PVT and PB-CeL projecting neurons. Control experiments were done with injections of the helper AAV and RVdG in the PB of rats that had not received the AAVrg-EBFP-Cre in the PVT since there is always a possibility that RVdG is transduced in neurons that lack Cre (Gehrlach et al. 2020; Miyamichi et al. 2013). We confirmed a lack of mCherry and HA labeling in PB neurons and a total absence of mCherry labeling in the entire brain. Additional controls were done with AAVrg-EBFP-Cre in the PVT without helper AAV in the PB followed by RVdG which show the same results as the no-Cre controls. These observations indicate that mCherry cells observed in the cases analyzed are indeed input cells to PB-PVT or PB-CeL projecting neurons.

### Intersectional anterograde tracing

**Fig. 14:**
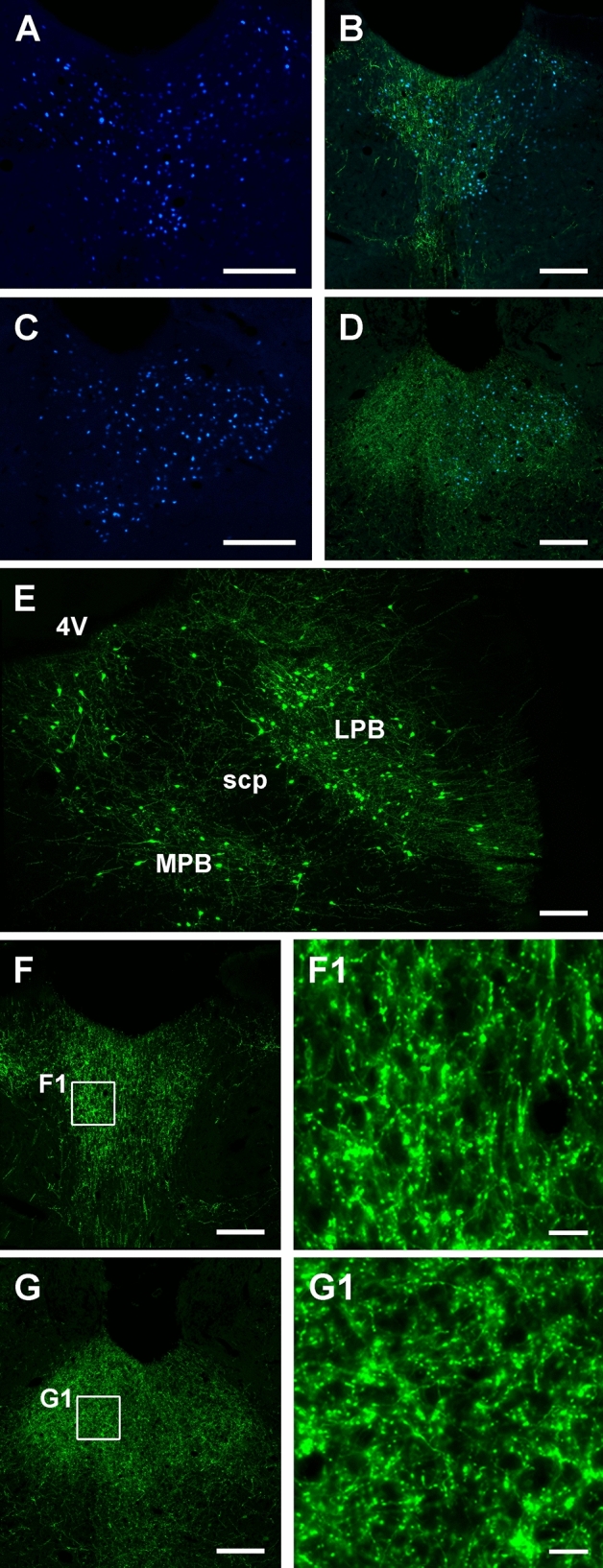
Collateral projections of PB-PVT neurons as shown using the intersectional anterograde tracing approach. Distribution of EBFP (blue) in the aPVT (**a**) and pPVT (**c**) produced by injections of the AAVrg-EBFP-Cre in the PVT with resulting GFP (green) anterograde fiber labeling overlapping the EBFP (blue) for the aPVT (**b**) and pPVT (**d**). Neurons were transduced in most regions of the PB but were concentrated in the LPB (**e**) producing dense GFP fiber labeling in the aPVT (**f**) and pPVT (**g**). Images **f1** and **g1** are higher magnification of the square inset in **f** and **g**, respectively. See list for abbreviations and numbers at the bottom represent distance from the bregma. Scale bars: 200 µm for **a**–**g**, 20 µm for **f1** and **g1**

**Fig. 15:**
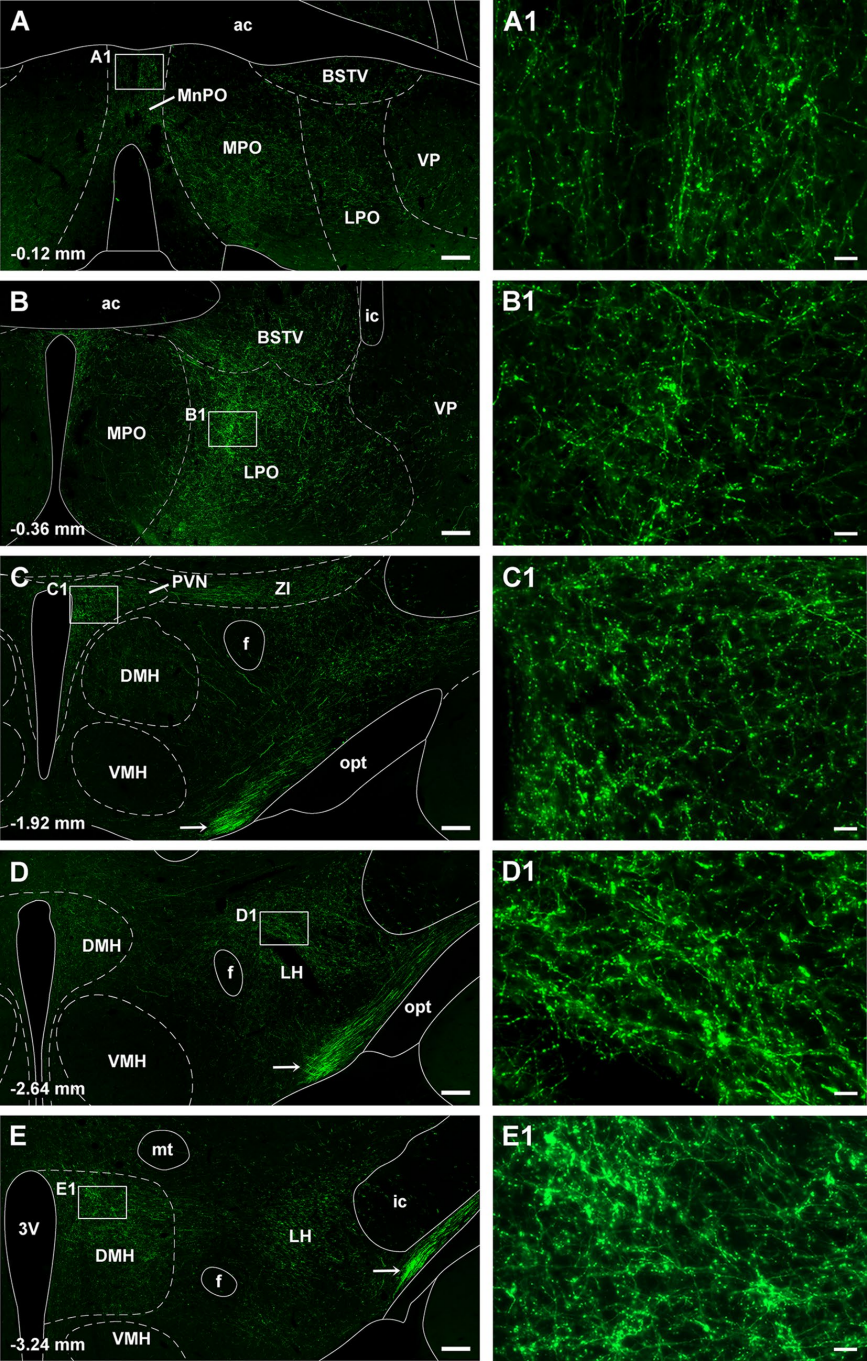
Images of the forebrain containing moderately dense GFP fiber labeling produced by the intersectional anterograde tracing approach applied to the PB-PVT neurons. Images of the GFP labeling produced by the case shown in Fig. 14 from the anterior to posterior levels of the forebrain (**a**–**e**). Images on the right are higher magnification of the square inset shown on the left. See list for abbreviations and numbers at the bottom represent distance from the bregma. Scale bars: 200 µm for **a**–**e**, 20 µm for **a1**–**e1**

**Fig. 16:**
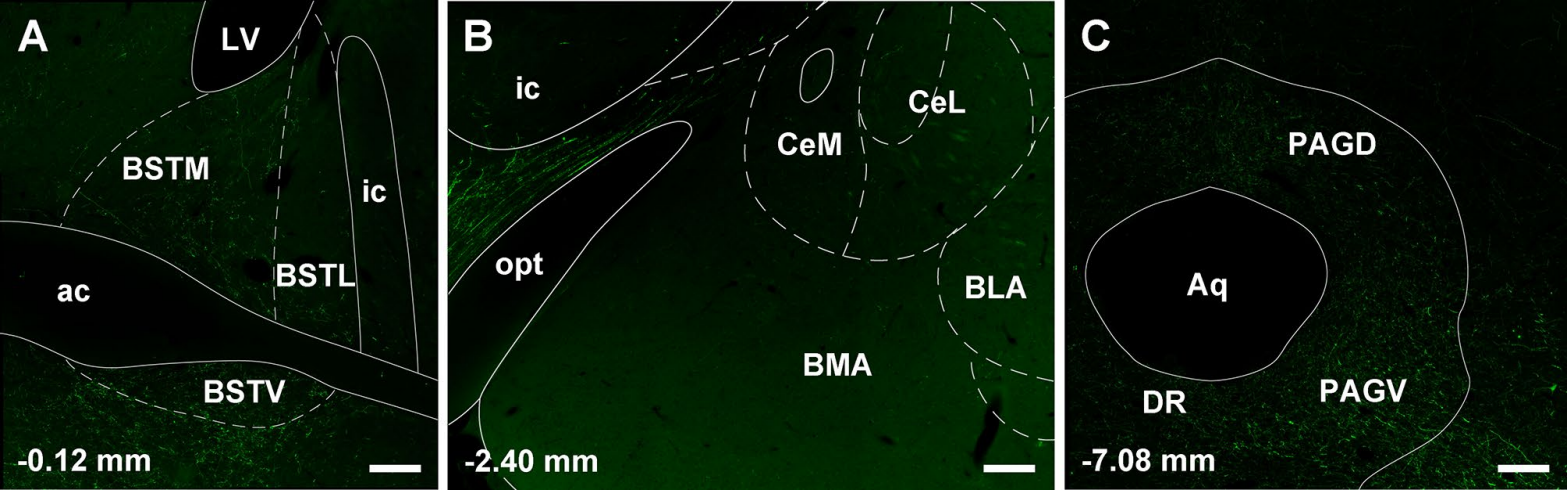
Images of brain regions lacking substantial GFP fiber labeling produced by the intersectional anterograde tracing approach applied to PB-PVT neurons. A relative lack of labeling was observed in the BSTDL (**a**), CeL (**b**) whereas the ventrolateral region of the PAG contained weak fiber labeling (**c**). See list for abbreviations and numbers at the bottom represent distance from the bregma. Scale bars: 200 µm

Injections of the AAVrg-EBFP-Cre were confined mainly to the aPVT and pPVT as evidenced by the reporter protein EBFP being confined to PVT neurons (Fig. 14A, C). These injections along with injections of AAV9/Flex-GFP above the superior cerebellar peduncle at the pontine-midbrain transition resulted in GFP expressing neurons mostly in the LPB in a pattern similar to what was observed in the TRIO tracing experiment (Fig. 14E). Transduced GFP fibers partially overlapped with the expression of EBFP in both the aPVT and pPVT indicating a point to point projection from the LPB to the PVT (Fig. 14B, D). A dense plexus of GFP fibers containing enlargements was observed bilaterally in both the aPVT and pPVT (Fig. 14F–G1). Other midline and intralaminar thalamic nuclei contained few GFP fibers (not shown). It is clear from examining the whole brain that PB-PVT neurons have axons that are highly collateralized because a number of areas in addition to the PVT contained a moderately dense plexus of GFP fibers. Fiber labeling was generally more robust on the ipsilateral side although the PVT receive a dense bilateral innervation. Cortical areas including the prefrontal cortex were devoid of fibers whereas the lateral septal area and the diagonal band of broca contained mostly scattered fibers. There was a moderate density of GFP fibers in the median preoptic nucleus (Fig. 15A, A1) and anterior aspect of the lateral preoptic area (Fig. 15B, B1) whereas the medial preoptic area was sparsely labeled (Fig. 15B). Fibers were seen in the zona incerta where they merged with a moderately dense fiber plexus found in the paraventricular nucleus of the hypothalamus (Fig. 15C, C1). The dorsomedial nucleus of the hypothalamus and perifornical lateral hypothalamic area contained a moderately heavy density of fibers (Fig. 15D–E1). Labeled fibers were observed crossing in the retrochiasm with a few of these fibers showing enlargements but with the majority appearing as smooth fibers of passage (arrow, Fig. 15C–E). Some regions of the brain were expected to contain GFP fibers based on previous description of the projections pattern provided by LPB neurons (Huang et al. 2021a). For instance, the BSTDL and CeL were almost completely devoid of fiber labeling except for a few scattered fibers (Fig. 16A, B). Ascending fibers of passage were observed in the substantia nigra, ventral tegmental area, retrorubral field, and pontine tegmentum as they extended toward the forebrain. Fibers ascending to the thalamus could also be seen passing along the cerebral aqueduct and the third ventricle whereas fiber labeling in the remainder of the brainstem was sparse except for a few regions. The dorsal and ventral columns of the PAG contained a moderate amount of fibers as did the pontine tegmentum (Fig. 16C). The medulla was largely devoid of labeled fibers except for light labeling in the raphe nuclei above the pyramidal tract (data not shown).

## Discussion

The results of the CTB tracing experiments are in line with previous studies reporting that a relatively small number of catecholamine neurons project to the PVT (Cornwall and Phillipson 1988; Chen and Su 1990; Krout et al. 2002, 2003; Krout and Loewy 2000a, b; Li and Kirouac 2012; Phillipson and Bohn 1994; Otake et al. 1994). The large number of neurons in the LPB compared to the rest of the brainstem is also consistent with our quantitative analysis of retrograde labeling in the brain following injections of CTB restricted to the aPVT and pPVT (Li and Kirouac 2012). It is generally understood that the PB relays brainstem and spinal cord viscerosensory, somato-sensory and taste-related signals to widespread regions of the forebrain (Chiang et al. 2019; Palmiter 2018; Herbert et al. 1990; Saper and Loewy 1980) including the PVT (Li and Kirouac 2012; Krout and Loewy 2000a). Mapping of brain-wide sources of neurons that make synapses on PB-PVT projecting neurons in the TRIO experiments revealed that the largest source of input cells is from neurons that are scattered in the brainstem RF. Other prominent sources of input cells include the PAG in addition to the superior and inferior colliculi of the midbrain tectum. Another notable observation from the rabies TRIO experiments is that the BSTDL and CeL are distinctive sources of afferents to PB-PVT neurons. The intersectional anterograde tracing approach demonstrates that PB neurons that project to the PVT do not innervate the BSTDL and CeL but provide collateral innervation to a number of forebrain regions postulated to regulate wakefulness, behavior and regulation of homeostasis. As discussed below, these anatomical findings provide evidence that the PVT is part of an interconnected network of neurons dispersed in the brainstem and forebrain that may regulate arousal and behavioral states.

### Lower brainstem inputs to the PVT

The PVT contains a relatively sparse plexus of small TH fibers (Takada et al. 1990; Otake and Ruggiero 1995) which has been shown to mostly originate from dopamine neurons in the hypothalamus and PAG (Li et al. 2014). The number and location of TH neurons in the lower brainstem that project to the aPVT and pPVT were assessed to further characterize the source of these fibers. A relatively small number of TH neurons confined to the C2/A2 cell group of the NTS and the C1/A1 cell group of the caudal VLM were found to project to the PVT as previously reported (Phillipson and Bohn 1994; Otake et al. 1994). The presence of TH indicates that these neurons produce either dopamine, norepinephrine or epinephrine depending on the presence of other rate-limiting enzymes (the nomenclature and function of the catecholamine cell groups are discussed in Bucci et al. 2017). Consistent with our previous retrograde tracing experiments (Li and Kirouac 2012; Li et al. 2014; Kirouac et al. 2006), there was a paucity of CTB cells in the LC even if a few LC-PVT projecting neurons has been reported by others (Krout et al. 2002; Otake and Ruggiero 1995). One likely explanation for this discrepancy may be that our approach of using fine glass pipettes to administer a small volume of CTB reduced the amount of the tracer picked up by damaged fibers coursing through the area. Viral transduction of LC neurons in dopamine β-hydroxylase-Cre mice provided evidence that the LC produces and releases dopamine in the PVT (Beas et al. 2018), but a functional LC projection to the PVT in the rat is difficult to envision considering that neuronal projections appear to be well conserved between rats and mice (Ellenbroek and Youn 2016).

The brainstem catecholamine cell groups and the nuclei with which they are associated are implicated in the viscerosensory reflexes and the execution of various behavioral and physiological responses to homeostatic challenges (Guyenet 2006; Guyenet et al. 2013; Ritter et al. 2006; Rinaman 2011). Consequently, it is possible that catecholamine signals may be integrated by the PVT to promote adaptive responses, as recently shown for hypoglycemia-induced food intake (Sofia Beas et al. 2020). However, the type of influence catecholamines have on PVT functions remains an open question for a number of reasons. First, catecholamine neurons that innervate the PVT are widely scattered in both the hypothalamus and brainstem and do not appear to form functionally distinct populations. Second, experimental evidence indicates that catecholamine neurons in the lower brainstem exert cellular effects predominately via conventional glutamatergic transmission (DePuy et al. 2013) and potentially the co-release of neuropeptides but not catecholamines (Guyenet et al. 2013). Third, the fact that brainstem catecholamine neurons form a meshwork of interconnected neurons that provide highly collateralized axons indicates that these neurons are likely to produce broad and diffused arousal effects on the brain including effect via extrasynaptic release and volume transmission (Aston-Jones and Cohen 2005; Bucci et al. 2017; Card et al. 2006; Guyenet et al. 2013; Patthy et al. 2021; Fuxe et al. 2010).

The PB is a multisensory relay center that is subdivided into medial and lateral regions that are further partitioned into cytoarchitecturally distinct subnulei (Chiang et al. 2019; Saper and Loewy 1980). It is clear from the present and our previous retrograde tracing experiments that the LPB is the area of the brainstem that contains the largest number of PVT projecting neurons (for quantitative analysis of the source of all brain inputs to the PVT, see Li and Kirouac 2012). We did not attempt to localize PB-PVT projecting neurons within subnuclear boundaries because of the difficulty of doing this with precision in thick sections stained with NeuN. Furthermore, dendrites of LPB neurons extend outside subnuclear boundaries making the relevance of these anatomical distinctions debatable (Sarhan et al. 2005; Jasmin et al. 1997). However, it is clear that CTB neurons were located in the general region of the LPB that contains the external, ventral and dorsal lateral subnuclei presumed to receive afferents from general viscerosensory integration regions of the NTS (Chiang et al. 2019; Saper and Loewy 1980; Herbert et al. 1990) and nociceptive signals from the brainstem and spinal cord (Chiang et al. 2019; Jaramillo et al. 2021; Han et al. 2015; Gauriau and Bernard 2002; Deng et al. 2020). Since most of the PVT projecting neurons were localized in the LPB, we will refer to PB neurons that project to the PVT as LPB-PVT projecting neurons for the remainder of the discussion with the caveat that some neurons in the MPB contribute to this projection.

### Whole brain inputs to LPB-PVT projecting neurons

The NTS was expected to be the major source of input cells to LPB-PVT neurons because of the dense projection between the NTS and the LPB (Herbert et al. 1990; Moga et al. 1990; Tokita et al. 2009). Nevertheless, we observed a low number of labeled cells in the NTS compared to the large number of input cells scattered broadly throughout the brainstem RF. This highlights the clear advantage of the rabies monosynaptic approach over traditional tracing methods that simply assess area-to-area projections. This is especially critical for LPB neurons which have dendrites that extend hundreds of micrometers (Sarhan et al. 2005). It should be appreciated that the large number of input cells in the RF represent cells that are distributed over a large area of neural tissue. The brainstem RF is conceptualized as a loosely organized network of interconnected neurons that integrate polymodal inputs to coordinate many vital functions (Blessing 1997). The RF is also postulated to be part of the ascending reticular activating system involved in the maintenance of arousal and behavioral states (Blessing 1997; Moruzzi and Magoun 1949; Liu and Dan 2019; Jones 2020; Scammell et al. 2017). While some regions of the RF are regarded as distinct nuclei with sensory or motor functions (Blessing 1997; Jones 1995), these regions were devoid of clusters of input cells. In terms of the NTS, input cells were mostly concentrated in the anterior portion of the NTS that receives gustatory afferents from the tongue in addition to overlapping viscero- and somato-sensory afferents from the oral cavity (Herbert et al. 1990; Lundry and Norgren 2015; Blessing 1997). Nonetheless, it is difficult to know the types of signals relayed to LPB-PVT neurons simply based on their location in the NTS (Herbert et al. 1990; Lundry and Norgren 2015; Blessing 1997). One possibility is that LPB-PVT neurons receive integrated polymodal sensory information from the oral cavity. A primary gustatory function is unlikely since LPB-PVT neurons were found in the non-gustatory regions of the PB (Blessing 1997; Chiang et al. 2019; Herbert et al. 1990). The parvicellular reticular nucleus of the medulla located adjacent to the anterior most portion of the NTS contained a distinctive collection of input cells. The parvicellular reticular nucleus processes signals related to sensory and motor functions associated with oral movements (Travers et al. 2000; Zerari-Mailly et al. 2001; Moriyama 1987; Takatori et al. 1981; Shammah-Lagnado et al. 1992) pointing to the possibility that the LPB-PVT neurons may receive signals related to ingestion. The absence of input cells in the nociceptive superficial lamina of the spinal trigeminal nucleus suggests that LPB-PVT neurons may not directly receive nociceptive signals from the head, but the likelihood that these neurons receive nociceptive signals from the spinal cord cannot be excluded from our experiments.

Input cells were scattered across multiple stereotaxic levels in a number of midbrain regions. These included the PAG, involved in the regulation of emotional and nociceptive responses (George et al. 2019; Behbehani 1995); the superior and inferior colliculi, implicated in the modulation of movements and arousal to visual and auditory stimuli (Brandao et al. 1994, 2003; Schenberg et al. 2005; Isa et al. 2021; Mulckhuyse 2018; Basso and May 2017; Cabrera et al. 2013); and the nucleus cuneiformis, linked to the modulation of locomotion and nociception (Coles et al. 1989; Jordan et al. 2008; Ebrahimzadeh and Haghparast 2011; Haghparast et al. 2010; Sandner et al. 1993; Bernard et al. 1989).

Several areas of the forebrain linked to the regulation of homeostasis and modulation of behavioral states contained a small number of input cells. First, input cells were found scattered in the median preoptic nucleus and the lateral preoptic area where some neurons display enhanced activity during sleep (McKinley et al. 2015; Szymusiak et al. 2007; Scammell et al. 2017). Sleep stimulates GABAergic neurons in the preoptic area (Gong et al. 2004) that promote sleep states by inhibiting arousal centers in the hypothalamus and brainstem (Szymusiak et al. 2007; Scammell et al. 2017). In addition to the regulation of sleep, the median preoptic nucleus is connected with areas of the hypothalamus involved in regulating body fluids, sodium, and temperature (McKinley et al. 2015; Menani et al. 2014). Input cells were also consistently observed in the LH, a functionally complex region that is involved in behavioral and physiological responses associated with survival including the regulation of sleep, arousal, energy balance, reward, stress and defensive responses (Berthoud 2002; Castro et al. 2015; Arrigoni et al. 2019; Scammell et al. 2017; Alexandre et al. 2013; Bonnavion et al. 2016).

Input cells were found as distinct clusters in the BSTDL, CeL, IPAC and parasubthalamic nucleus. The BSTDL, CeL and IPAC form a macrostructure called the extended amygdala that plays a critical role in adaptive responding (de Olmos et al. 2004; Alheid 2003; Alheid et al. 1999). The parasubthalamic nucleus is interconnected with the BSTDL, CeL, IPAC and LPB; and may be part of a network of neurons involved in mediating integrated behavioral and physiological responses (Goto and Swanson 2004; Shah et al. 2021) as recently demonstrated for fear-induced hypothermia (Liu et al. 2021a). The parasubthalamic nucleus also projects to the pPVT (Goto and Swanson 2004) and regions of the prefrontal cortex that in turn innervate the pPVT and the extended amygdala (Li and Kirouac 2012). The BSTDL/CeL are best known for their role in regulating responses to threats (Lebow and Chen 2016; Gungor and Pare 2016; Duvarci and Pare 2014; Fox et al. 2015; Davis and Shi 1999), potentially in response to feedforward signals from the LPB about aversive conditions (Han et al. 2015; Phua et al. 2021; Cai et al. 2018; Jaramillo et al. 2020; Ito et al. 2021; Bowen et al. 2020; Chiang et al. 2020) and the PVT (Do-Monte et al. 2017, 2015; Penzo et al. 2015; Pliota et al. 2018). Subpopulations of GABA neurons in the BSTDL/CeL express unique neurochemical markers that differentially contribute to defensive responses through mechanisms and projections that are not completely understood (Gungor and Pare 2016; Duvarci and Pare 2014; Fadok et al. 2018; Li 2019). Regardless of neuropeptides associated with these neurons, they are likely to have inhibitory effects on LPB-PVT neurons because GABA is the primary neurotransmitter associated with these projection neurons (Moga and Gray 1985; Ye and Veinante 2019; Bartonjo and Lundy 2020). Although we did not identify the neuropeptide associated with the neurons in the BSTDL/CeL that synapse on LPB-PVT neurons, corticotropin-releasing factor, somatostatin, and neurotensin are potential candidates as these markers have been shown to be associated with some BSTDL/CeL GABAergic neurons that project to the LPB (Moga and Gray 1985; Ye and Veinante 2019; Bartonjo and Lundy 2020). Future investigation on the identity and activation conditions of BSTDL/CeL neurons that synapse on LPB-PVT neurons would yield critical information about how these long-range circuits function in the brain.

The lateral substantia nigra (SNL) is another brain region found to be a distinct source of input to LPB-PVT neurons. This group of cells is of special interest because it receives afferent input from the BSTDL/CeL (Steinberg et al. 2020; Gonzales and Chesselet 1990; Vankova et al. 1992; Liu et al. 2021b) and a projection from the CeL to the SNL has been shown to modulate both appetitive and aversive learning (Steinberg et al. 2020). Furthermore, the SNL and LPB are reciprocally connected (Tokita et al. 2009; Huang et al. 2021a; Vankova et al. 1992) forming potentially an interconnected network of neurons involving the PB, PVT, SNL and BSTDL/CeL. Likewise, the LPB may also indirectly send neural signals to the BSTDL/CeL via projections to the PVT and parasubthalamic nucleus (Li and Kirouac 2012; Huang et al. 2021a, 2021b) which in turn densely innervate the BSTDL/CeL (Li and Kirouac 2008; Goto and Swanson 2004) forming another interconnected network involving the LPB, PVT and the BSTDL/CeL.

The location and density of input cells observed in the TRIO experiments were generally consistent with what has been reported in rats and mice following injections of retrograde tracers involving much of the PB (Moga et al. 1990; Tokita et al. 2009; Herbert et al. 1990). However, there were notable exceptions including the absence of input cells from prefrontal cortical areas and medial region of the central nucleus of the amygdala. The large number of input cells in the CeL combined with the absence of such cells in the medial part of the central nucleus attests to the specificity of the synaptic connections associated with a CeL-PB-PVT circuit. However, it is possible that some input areas were not identified since monosynaptic transport of rabies has been shown to be ineffective for some projection systems (Beier 2019). It is clear that the BSTDL/CeL synaptic inputs to LPB neurons are not unique to neurons that project to the PVT since the TRIO approach applied to PB neurons that project to the CeL also resulted in distinctive clusters of input cells in the BSTDL/CeL. The sources of inputs to PB-PVT and PB-CeL neurons were similar except for the reticular portion of the substantia nigra that appeared to preferentially synapse on PB-CeL neurons. The reticular portion of the substantia nigra is a basal ganglia output nucleus involved in the regulation of motor and cognitive responses via thalamocortical feedback circuits (Shipp 2017; Alexander et al. 1990; Deniau et al. 2007). Input cells in the reticular substantia nigra that synapse on PB-CeL may represent a newly identified feedback circuit from the basal ganglia to the PB neurons that innervate the CeL and potentially the BSTDL.

### Whole brain projections of LPB-PVT neurons

Anterograde tracing of the collateral innervation pattern of LPB-PVT neurons indicates that these neurons are largely specific to the PVT although retrograde tracing studies indicate that other midline thalamic nuclei receive input from the LPB (Krout et al. 2002; Krout and Loewy 2000a). The present paper and other recent evidence support the view that some neurons in the PB preferentially innervate unique thalamic nuclei (Deng et al. 2020; Huang et al. 2021a). Glutamatergic neurons in the LPB that co-express the neuropeptides corticotropin-releasing factor (CRF), substance P, cholecystokinin (CCK) and cocaine- and amphetamine-regulated transcript (CART) project to the PVT (Kirouac et al. 2006; Otake and Nakamura 1995; Otake 2005). Experimental approaches targeting marker-specific neurons have been used to identify subpopulations of function- and projection-specific neurons in the PB. There are a number of examples in which the evidence indicates that subpopulations of LPB neurons that express particular peptides may be differentially activated by particular homeostatic challenges including nociception, body temperature, taste stimuli, hypoxia and other life threatening states (see reviews Jaramillo et al. 2021; Chiang et al. 2019; Palmiter 2018). For example, neurons that express calcitonin gene-related peptide (CGRP) have been shown to be activated by most threats examined (Carter et al. 2013; Han et al. 2015; Palmiter 2018) and to project robustly to the BSTDL and CeL (Huang et al. 2021b). However, it is apparent from our anterograde tracing experiments that LPB neurons that innervate the PVT are different from the population that projects to the BSTDL/CeL. Neurons that express dynorphin have been shown to respond to changes in ambient temperatures and to have projection patterns different from the CGRP cells (Geerling et al. 2016; Huang et al. 2021a). Other research groups have shown that dynorphin neurons in the LPB respond to a variety of somato-sensory signals including nociception relayed from local CGRP neurons (Choi et al. 2020; Kim et al. 2020; Luskin et al. 2021; Chiang et al. 2020). Glucose-sensing PB neurons that express CCK were shown to project to the ventromedial nucleus of the hypothalamus but not the PVT (Garfield et al. 2014). No attempt was made here to identify the neuropeptide identity of LPB-PVT neurons because of the complexity in the number of neurons in the PB that express one or more peptides (Palmiter 2018; Chiang et al. 2019; Zhu et al. 2022). Regardless if LPB-PVT neurons have a specific neuropeptide genotype, PB neurons will likely exert excitatory effects on the PVT via the release of glutamate (Huang et al. 2021a; Geerling et al. 2017; Niu et al. 2010; Zhu et al. 2022). Nevertheless, application of single-cell sequencing to categorize unique genes highly expressed in LPB-PVT neurons will be helpful for examining the function of these neurons.

The collateral projections provided by LPB-PVT neurons hint at potential functions associated with these neurons. Areas receiving most significant collateral innervation included the preoptic area, zona incerta, LH, and dorsomedial nucleus of the hypothalamus (DMH). Many of these regions are associated with arousal and the regulation of behavioral states. The zona incerta is postulated to be part of a larger brain-wide interconnected network involved in modulation of arousal, attention, and movements including approach and avoidance to interoceptive and exteroceptive sensory information (Mitrofanis 2005). It is notable that the zona incerta is interconnected with many of the same midbrain regions (Kolmac et al. 1998) shown in the TRIO experiments to make synapses on LPB-PVT neurons. The paraventricular nucleus of the hypothalamus is a key nucleus involved in the regulation of the autonomic nervous system and the stress response (Dampney 1994; Myers et al. 2014). The DMH is linked to the modulation of circadian and biological rhythms in addition to contributing to the physiological and hormonal responses to threats (DiMicco et al. 2002; Fontes et al. 2011; Saper et al. 2005; Myers et al. 2014). As discussed above, the preoptic area and LH are brain regions that are broadly associated with arousal and the modulation of homeostasis and behavioral states. It is of potential significance that some forebrain regions that contain neurons that make monosynaptic connections with LPB-PVT neurons are the same regions that received significant collateral fiber innervation from LPB-PVT neurons (e.g., LH). While we demonstrate that neurons in the preoptic area and LH make synaptic connections with LPB-PVT neurons, we do not know if fibers from LPB-PVT neurons make synaptic connections with neurons in these reciprocally innervated regions. The fact that the median preoptic nucleus, DMH, and LH project to the PVT (Kirouac et al. 2005; Li and Kirouac 2012; Thompson et al. 1996; Goto and Swanson 2004) further highlighting the possibility that neurons in the PVT may be part of a larger network of neurons with broadly similar functions.

### Functional considerations

This paper presents anatomical evidence that demonstrates that the LPB is the major source of lower brainstem afferents to the PVT. The LPB has received considerable interest for its role in regulating behavioral and physiological responses to exteroeceptive and interoceptive signal related to homeostatic challenges (Palmiter 2018; Jaramillo et al. 2021; Chiang et al. 2019). The fact that the LPB is considered a major integrating and relay center of spinal cord and brainstem sensory signals to forebrain led to the search for projection-specific neurons in the LPB that regulate responses to specific challenges. For instance, arousal from sleep during hypercapnia (high CO_2_) was shown to be mediated by CGRP neurons that act on circuits primarily located in the basal forebrain and to a lesser extent in the LH and CeL (Kaur et al. 2017). Furthermore, neurons that express CGRP also respond to nociception associated with footshocks and conditions known to suppress appetite; and optogenetic activations of these projections to the CeL diminish food intake (Carter et al. 2013) and produce an aversive teaching signal that results in a retrievable fear memory (Han et al. 2015). Neurons in the LPB send highly collateralized fiber projections to interconnected sites in the brain (Sarhan et al. 2005; Bowen et al. 2020; Chiang et al. 2020; Zhu et al. 2022) and attempts at isolating specific defensive behavioral and physiological responses to one anatomical terminal field have proven difficult using optogenetic approaches (Bowen et al. 2020; Chiang et al. 2020). The difficulty in assigning specific functions to subpopulations of PB neurons is compounded by the fact that these neurons exist as intermixed populations and not distinct clusters of cells (Huang et al. 2021a, 2021b; Bowen et al. 2020; Barik et al. 2018; Chiang et al. 2020). The latter is further complicated by the fact that neurons in the PB have dendrites and axons that extend across subnuclear zones (Sarhan et al. 2005; Jasmin et al. 1997; Chiang et al. 2020). Another notable observation made in the present paper is that the LPB neurons that innervate the PVT do not project to the BSTDL/CeL. Indeed, our understanding of how the PB integrates and relays signals to other regions of the brain is preliminary and limited by the complexity of the types of neurons that make up the PB including their unique afferent and efferent connections.

The number and location of input cells that synapse on LPB-PVT neurons provide some indications of the type of information these neurons broadcast to the PVT. The fact that major sources of input originate from neurons scattered in brainstem regions functionally associated with general arousal suggests that LPB-PVT projecting neurons may be part of an ascending arousal system. Inputs from the superior and inferior colliculi also indicate that LBP-PVT neurons may integrate signals linked to arousing visual and auditory stimuli. It is generally appreciated that arousal and the maintenance of wakefulness are mediated by a highly distributed network of interconnected neurons that span the forebrain, midbrain, and hindbrain (Liu and Dan 2019; Jones 2020; Scammell et al. 2017). The PB has been identified as a critical brainstem structure involved in arousal from sleep via connections to the basal forebrain and the LH (Venner et al. 2019; Qiu et al. 2016; Fuller et al. 2011; Xu et al. 2021; Kaur et al. 2013, 2017). While there is some evidence that the PVT is involved in arousal from sleep (Hua et al. 2018; Matyas et al. 2018; Ren et al. 2018), other evidence indicates that the thalamus is not critical for the PB’s cortical arousal effects (Qiu et al. 2016; Fuller et al. 2011; Xu et al. 2021).

General arousal has also been conceptualized as a brain activation phenomenon that invigorates behavior by enhancing the activity of the sensory and motor circuits (Pfaff et al. 2008; Pfaff 2006). Accordingly, another possibility is that general arousal signals enhance the activity of PVT neurons thereby invigorating behavioral responses. It should also be appreciated that factors that promote behavioral responses have general arousal effects on the brain (Pfaff 2006). Determinants of behavior such as physiologic states (e.g., hunger, thirst, nociception, pheromones) and cues that signal potential appetitive and aversive outcomes are often reported to increase the activity of PVT neurons (for details and references, see Barson et al. 2020; Millan et al. 2017; Hsu et al. 2014; Kirouac 2015, 2021; Penzo and Gao 2021). We postulate that PVT neurons integrate incoming signals that sense the arousal state of the brain with neural inputs signaling the presence of physiologic states and emotionally salient cues. For instance, the PVT uses hypothalamic and brainstem signals related to the energy and hydration to modulate behavioral responses to maintain homeostasis (Livneh et al. 2017; Zhang and van den Pol 2017; Hua et al. 2018; Sofia Beas et al. 2020; Leib et al. 2017; Labouebe et al. 2016; Zhu et al. 2018; Meffre et al. 2019). The PVT also receives signals related to predator odor via the ventromedial nucleus of the hypothalamus to suppress food reward seeking (Engelke et al. 2021). Other evidence shows that the PVT integrates signals related to emotionally salient cues from the prefrontal cortex to modulate behavioral responses (Otis et al. 2019, 2017; Do-Monte et al. 2015; Penzo et al. 2015). The type of influence that the PVT has appears to be related to whether a response poses a threat or has a chance of being unproductive supports the view that the PVT is also involved in the selection of responses based on the integration of a wide range of afferent inputs (Cheng et al. 2018; Do-Monte et al. 2017; Lafferty et al. 2020; Choi et al. 2019; Choi and McNally 2017). As a whole, various experimental and anatomical observations suggest that the PVT integrates a broad range of signals related to saliency of cues, homeostatic state, and general arousal to modulate behavioral responses that promote survival.

**Fig. 17:**
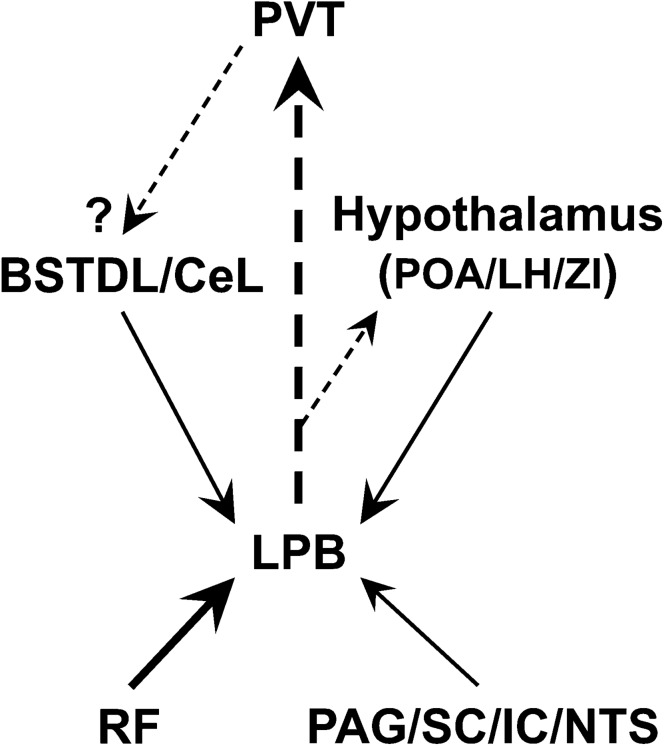
Schematic diagram summarizing some of the more salient anatomical findings from the TRIO and anterograde tracing approaches. The size of the arrows indicates the strength of the projection whereas the arrow with the dashed line indicates the lack of evidence for synaptic connectivity. See list for abbreviations

Figure 17 summarizes some of the more salient anatomical findings reported in this paper. As discussed above, the number of neurons scattered in the brainstem RF that provide synaptic input to the LPB-PVT neurons is especially noteworthy. In addition, a considerable proportion of inputs originate from neurons located in the NTS, PAG, tectum, and hypothalamus. An intriguing source of inputs originates from clusters of neurons in the BSTDL and CeL, areas of the extended amygdala also densely innervated by the PVT (Dong et al. 2017; Li and Kirouac 2008) and known to modulate behavioral responses to threats (Gungor and Pare 2016; Duvarci and Pare 2014; Fadok et al. 2018; Li 2019). The BSTDL/CeL could be part of a feedback circuit that influences the transmission of a variety of signals from the LPB to the PVT (i.e., gating mechanism). It is not known if PVT neurons make synaptic contact with neurons in the BSTDL/CeL that synapse on the LPB-PVT neurons nor do we know if the PVT neurons that project to the BSTDL/CeL receive synaptic contacts from the LPB neurons. Nonetheless, this is an intriguing possibility as it would provide a means by which a multisynaptic circuit could modulate the flow of arousal and/or other homeostatic signals to the PVT and other areas of the forebrain. In support, a recent paper identified projections from the BSTDL that modulate threat assessment and feeding by acting on PB neurons (Luskin et al. 2021). Both the LPB and PVT are areas of the brain often reported to be activated during aroused and emotional states (Hsu et al. 2014; Kirouac 2015; Palmiter 2018; Jaramillo et al. 2021; Chiang et al. 2019). Optogenetic excitation of PB fibers in the PVT and chemogenetic activation of PB-PVT neurons elicit responses indicative of an aversive state (Zhu et al. 2022). However, these findings should be interpreted cautiously since these manipulations are likely to have produced activation of most of the collaterals associated with these neurons and not only those projecting to the PVT. Our results also present the intriguing possibility that a descending GABAergic projection from the BSTDL/CeL (Moga and Gray 1985; Ye and Veinante 2019) could dampen the excitatory effects of arousal-related signals on LPB-PVT neurons. Effective modulation of excitatory inputs could be profound if descending GABAergic inputs synapse on the soma or proximal dendrites of LPB-PVT neurons (Hao et al. 2009; Kandel and Siegelbaum 2000). Such a multisynaptic circuit could provide a mechanism by which the BSTDL/CeL could gate ascending signals related to homeostatic state of the body to the PVT and other areas of the forebrain. Changes in this circuitry could lead to maladaptive hyperaroused states and dysregulation of homeostasis.

## Data Availability

Available with reasonable request.

## Abbreviations

3 V: Third ventricle
4 V: Fourth ventricle
7 N: Facial nucleus
ac: Anterior commissure
aq: Aqueduct
BLA: Basolateral amygdala
BMA: Basomedial amygdala
BSTDL: Bed nucleus of stria terminalis, dorsolateral
BSTL: Bed nucleus of stria terminalis, lateral
BSTM: Bed nucleus of stria terminalis, medial
BSTV: Bed nucleus of stria terminalis, ventral
BSTVL: Bed nucleus of stria terminalis, ventrolateral
CeL: Central amygdala, lateral-capsular division
CeM: Central amygdala, medial
CnF: Nucleus cuneiformis
cp: Cerebral peduncle
CPu: Caudate putamen
CTB: Cholera toxin subunit B
DMH: Dorsomedial hypothalamus
DR: Dorsal raphe
dscp: Decussation superior cerebellar peduncle
DT: Dorsal tegmental nucleus
f: Fornix
Hb: Habenular nucleus
ic: Internal capsule
IC: Inferior colliculus
IMD: Intermediodorsal nucleus of thalamus
IPAC: Interstitial nucleus of the posterior limb of the anterior commissure
KF: Killiker-Fuse nucleus
LC: Locus ceruleus
LH: Lateral hypothalamus
LPB: Lateral aspect of the parabrachial nucleus
LPO: Lateral preoptic area
LRt: Lateral reticular nucleus
LV: Lateral ventricle
MD: Mediodorsal nucleus of thalamus
MdRt: Medullary reticular nucleus
Me5: Mesencephalic trigeminal nucleus
mfb: Medial forebrain bundle
MGN: Medial geniculate nucleus
ml: Medial lemniscus
MnPO: Median preoptic nucleus
MPB: Medial aspect of the parabrachial nucleus
MPO: Medial preoptic nucleus
Mt: Mammillothalamic tract
NAcSh: Shell of the nucleus accumbens
NTS: Nucleus of the solitary tract
opt: Optic tract
PAG: Periaqueductal gray
PAGD: Periaqueductal gray, dorsal
PAGV: Periaqueductal gray, ventral
PB: Parabrachialc nucleus
PCRt: Parvicellular reticular nucleus
PLH: Perifornical lateral hypothalamus
POA: Preoptic area
PSTh: Parasubthalamic hypothalamus
PT: Paratenial nucleus of the thalamus
PVN: Paraventricular nucleus of the hypothalamus
PVT: Paraventricular nucleus of the thalamus
aPVT: Anterior aspect of the paraventricular nucleus of the thalamus
pPVT: Posterior aspect of the paraventricular nucleus of the thalamus
py: Pyramidal tract
RF: Reticular formation
mRF: Mesencephalic reticular formation
mdRF: Medullary reticular formation
pRF: Pontine reticular formation
rs: Rubrospinal tract
SC: Superior colliculus
scp: Superior cerebellar peduncle
scpd: Superior cerebellar peduncle, descend limb
sm: Stria medullaris of the thalamus
SNL: Lateral substantia nigra
sp5: Spinal trigeminal tract
Sp5: Spinal trigeminal nucleus
Sp5C: Spinal trigeminal nucleus, caudal
STh: Subthalamic hypothalamus
TH: Tyrosine hydroxylase
TRIO: Tracing the input–output organization method
Ve: Vestibular nucleus
VLM: Ventrolateral medulla
VMH: Ventromedial hypothalamus
VP: Ventral pallidum
ZI: Zona incerta
